# Nickelferrite on Pnictogen Functionalized Carbon Nanohorns. Bifunctional ORR and OER Catalysts in Rechargeable Zinc‐Air Batteries

**DOI:** 10.1002/advs.77749

**Published:** 2026-09-27

**Authors:** Sudheer Kumar Yadav, Vanessa Trouillet, Jonas Lins, Torsten Gutmann, Jörg J. Schneider

**Affiliations:** ^1^ Department of Chemistry Inorganic Chemistry Eduard‐Zintl‐Institut Für Anorganische und Physikalische Chemie Technical University Darmstadt Darmstadt Germany; ^2^ Institute For Applied Materials (IAM‐ESS) and Karlsruhe Nano Micro Facility (KNMFi) Karlsruhe Institute of Technology (KIT) Eggenstein‐Leopoldshafen Germany; ^3^ Eduard‐Zintl‐Institute for Inorganic and Physical Chemistry Technical University Darmstadt Darmstadt Germany; ^4^ Department of Chemistry Physical Chemistry, Paderborn University Paderborn Germany

**Keywords:** bifunctional catalysts, nickel ferrite, nitrogen, phosphorus‐co doping, oxygen evolution reaction, oxygen reduction reaction, zinc‐air battery

## Abstract

In Nickel ferrites a low oxygen reduction activity and poor stability represent a drawback in rechargeable zinc‐air battery technology. This contribution presents a strategy using self‐templating for fabricating a nanohybrid material composed of NiFe_2_O_4_ nanocubes encapsulated in N, P‐doped carbon nanohorns (CNH). Benefiting from the efficient mass and electron transfer of such N, P‐doping, CNH provide a significant number of active sites preventing aggregation and dissolution of active ferrite components in the composite. Thus, the NiFe_2_O_4_@CNH‐N‐P nanohybrid catalyst exhibits a high half‐wave potential (0.79 V) and a low Tafel slope of 56.5 mV/dec, following a four‐electron pathway for oxygen reduction (ORR). The NiFe_2_O_4_@CNH‐N‐P nanohybrid catalyst work in full‐cell zinc air battery configuration as bifunctional cathode catalyst in conjunction with a ZnO/C composite anode. The maximum power density derived from polarization curves for ZAB based on NiFe_2_O_4_@CNH‐N‐P is 54 mW/cm^2^ and thus exceeds a benchmark catalyst composed of 20% Pt/C+RuO_2_ of 28 by 26 mW/cm^2^. The assembled secondary ZAB can be cycled for more than 300 h in a galvanostatic charge–discharge test. This work provides insight into the rational composition and morphology design, as well as full materials and electrochemical characterization, of an earth‐abundant electrocatalyst with efficient electrocatalytic activity.

## Graphical abstract

A systematic study on the performance of N,P‐doped NiFe_2_O_4_@CNH bi‐functional hybrid electrocatalysts for rechargeable zinc‐air batteries is presented. The importance of P, N‐doping and the intimate tethering of nickel‐ferrite nanocubes on the carbon nano‐horn (CNH) surface which are well dispersed on the surface of N,P doped CNHs. The urea‐assisted precursor methodology is versatile and can be extended to the synthesis of a broad range of mixed‐metal oxide‐based catalysts.

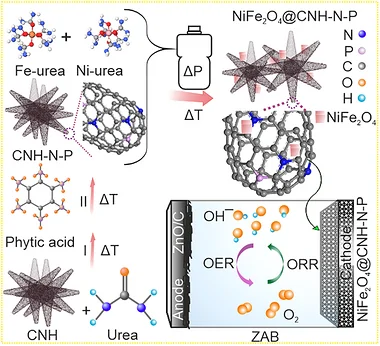

## 1 Introduction

The exhaustion of conventional fossil‐fuel energy resources and their environmentally unfriendly nature have created an urgent need for efficient electrochemical energy storage and transition technologies [1, 2]. Rechargeable zinc–air batteries (ZABs) are promising next‐generation energy storage devices due to their high energy density, environmental safety, and available zinc resources [3]. The ZAB's efficiency depends largely on the oxygen reduction reaction (ORR) and the oxygen evolution reaction (OER) at the air cathodes. Thus, effective oxygen electrocatalysts capable of promoting both ORR and OER, are essential for the efficient operation of rechargeable ZABs. Noble metal catalysts (Pt and RuO_2_/IrO_2_) are not adequate due to insufficient durability, high overpotentials, and high cost [4]. On the other hand, inexpensive nanohybrids of transition metal oxides and heteroatom‐doped carbon have attracted significant research attention for their promising bifunctional catalytic activity, making them promising candidates for ZABs.

Transition metal oxides and their ferrites are interesting materials due to their water oxidation activity, plentiful reserves, and adjustable electronic structures. Among transition‐metal ferrites, NiFe_2_O_4_, a bimetallic oxide with a spinel ABO_4_ structure, exhibits good catalytic activity and corrosion resistance. It possesses outstanding ORR/OER activity potential due to the presence of a high number of oxygen vacancies and redox couples Ni^2+^/Ni^3+^, Fe^2+^/Fe^3+^ [5]. Such abundant redox couples could provide a large number of active sites for adsorption, desorption, and the electronic transformation of reaction intermediates. Electronic exchange at octahedral sites within cations in variable ionic states significantly enhances electron transfer capability [5]. However, to the best of our knowledge, only a few reports describe nickel ferrite‐based nanohybrids with bifunctional applications in zinc‐air batteries [6, 7, 8, 9, 10, 11, 12, 13]. This can be traced to its semiconducting nature, which exhibits low electrical conductivity, and hinders essential electron transfer, resulting in inefficient utilization of the active sites. Moreover, during electrocatalytic activity, nickel ferrite undergoes volume disparities, leading to weakened structural stability. Providentially, the problem of structural instability and low electronic conductivity of nickel ferrite can be solved by morphology engineering (nano‐cubes, nanotubes, and nanospheres) and by creating nanohybrids with heteroatoms (P, S, N, etc.) doped carbon nanostructures (CNTs, graphene, carbon fibres, etc) [14, 15]. With respect to heteroatom doping in a carbon framework, dopants with different electronegativities help to polarize the adjacent carbon atoms, leading to improved charge and mass transfer. A carbon framework doped with two different dopant atoms shows superior electroactivity compared to a mono‐doped, exclusively due to the synergetic effect among the hetero dopant atoms [16, 17].

Nanostructured carbon materials as catalyst supports, such as graphene, carbon nanotubes (CNTs), and carbon fibres, have been extensively studied over the past few decades due to their good accessibility, high surface areas, and environmentally benign characteristics. However, reports on the use of carbon nanohorns (CNH) in the rapidly developing field of electrocatalysis remain very limited. In the present study, CNHs were selected over traditional supports such as CNTs or graphene due to their unique structural and practical merits. Crucially, CNH are synthesized via laser ablation or arc discharge of graphite, yielding high‐purity nanostructures (>95% purity) [18]. Structurally, individual CNHs naturally group into 3D ‘dahlia‐like’ spherical clusters. While pristine nanohorns possess closed conical tips, the topological strain at these tips allows them to readily open during subsequent chemical treatment or heteroatom doping. This tip‐opening provides access to their inner hollow cavities, making both internal and external surface areas far more accessible than the enclosed inner walls of CNTs [19]. The topological strain allows better accessibility for N, P‐doping in CNH. Furthermore, the mesoporous channels formed between neighboring nanohorns prevent the severe π‐π restacking observed in graphene, thereby ensuring rapid electrolyte penetration and O_2_ transport during ORR/OER [20]. Structurally, CNHs are typically composed of single tubules of carbon layers with a length of 30–50 nm and a diameter of 2–3 nm. They are aggregated due to strong inter‐tubular van der Waals interactions forming spherical bundles of “dahlia‐like” and “bud‐like” arrangements. Their typical diameter is between 80 and 100 nm [19, 21]. Electrocatalytic activities of nitrogen‐functionalized CNH, as well as on their use as supports in bifunctional nanohybrids, are, however, rare [14, 22]. To the best of our knowledge, there are only a few reports on the electroactivity of nitrogen‐ and phosphorus‐functionalized/co‐doped CNH (viz., CNH‐N‐P), and none on their use as a support in bifunctional nanohybrids, and any application‐based research in secondary, rechargeable ZABs are unknown [21, 23].

Herein, we report on a novel methodology for the creation of spinel NiFe_2_O_4_ nanocubes anchored on N‐ and P‐functionalized carbon nanohorns (NiFe_2_O_4_@CNH‐N‐P) as an efficient bifunctional cathode catalyst for ZABs. Initially, CNHs were pre‐functionalized in a stepwise process: the first step is N‐functionalization via heat treatment of a uniform mixture of CNH and urea to obtain N‐CNH; the second step is P‐functionalization of N‐CNH via hydrothermal treatment with phytic acid, followed by heat treatment to obtain CNH‐N‐P. Afterward, a NiFe_2_O_4_@CNH‐N‐P nanohybrid was obtained by hydrothermal treatment of a stoichiometric mixture of molecular precursors based solely on nickel urea nitrate (Ni‐urea) and iron urea nitrate (Fe‐urea) complexes, mixed with pre‐N‐P functionalized CNH (CNH‐N‐P). Pristine CNH exhibits two‐electron reaction kinetics for the oxygen reduction reaction [24], however, after functionalization and heteroatom doping, it shifts toward four‐electron reaction kinetics, clearly indicating the high activity of the tethered nickel ferrite species as active catalyst sites.

The elements N, P, C, Ni, and Fe are earth‐abundant, thereby enabling NiFe_2_O_4_@CNH‐N‐P nanohybrids a cost‐effective material. The redox couple Ni/Fe induces chemical coupling effects, achieving a form of synergism between both elements and resulting in increased catalytic performance. Conversely, the NiFe_2_O_4_ anchored in the CNH matrix inhibited its accumulation and uncovered additional electrocatalytically active sites. Moreover, the P, N‐functionalized CNH serves as excellent structural support and may offer efficient charge‐transfer channels, further enhancing the overall electrocatalytic performance. This synergistic combination leads to efficient electrocatalytic activity of NiFe_2_O_4_@CNH‐N‐P. It exhibits comparable overpotential, a lower Tafel slope, and greater stability than the standard catalyst (20% Pt/C + RuO_2_). The work presents a novel strategy for designing high‐performance, sustainable catalysts based on molecular precursors and a co‐doped CNH support. The presented approach not only enhances individual ORR and OER efficiencies but also delivers significant performance gains when used as a bifunctional catalyst in secondary ZABs. Such a development could assist in the growth of green energy technologies.

## 2 Results and Discussion

### 2.1 Catalyst Synthesis and Characterization

**SCHEME 1:**
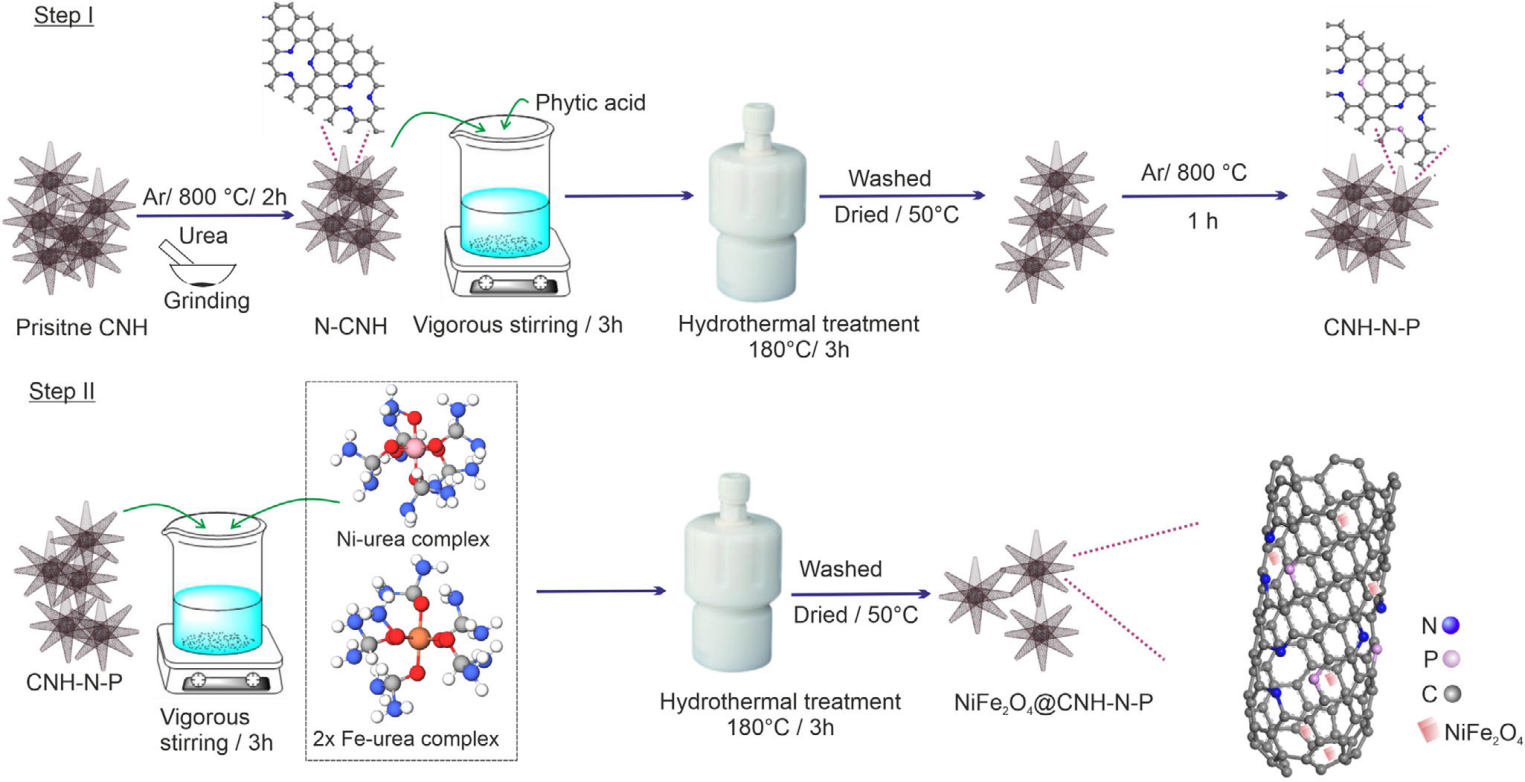
Schematic representation demonstrating the two‐step process to obtain the NiFe_2_O_4_@CNH‐N‐P catalyst material. Step I: N, P‐functionalization of CNH. Step 2: Hydrothermal synthesis of the final nanohybrid NiFe_2_O_4_@N‐CNH‐N‐P employing the homoleptic nickel and iron urea complexes as molecular precursors.

Nickel urea nitrate [Ni(NH_2_CONH_2_)_6_(NO_3_)_2_] and iron urea nitrate [Fe(NH_2_CONH_2_)_6_(NO_3_)_3_], molecular precursors are prepared by a precipitation method. FT‐IR analysis confirms the integrity of precursor complexes (Figure S1a) [25]. Thermogravimetric analysis (TGA) of a 1:2 (Ni‐urea and the Fe‐urea) mixture in an oxygen atmosphere provides insight into a suitable decomposition approach that could lead to the formation of nickel ferrite. Decomposition of this mixture commences at 129°C with a 78% mass loss up to 210°C. Thereafter, a 7% loss up to 430°C, after which the definitive mass attains 14.0%. Hence, the significant decomposition step of the precursor complex mixture occurs around 130–210°C, and the ultimate single product obtained is NiFe_2_O_4_ (see XRD pattern of remnant after TGA, Figure S1b). Based on these results, we designed the hydrothermal reaction to obtain a morphologically stable NiFe_2_O_4_@CNH‐N‐P nanohybrid as a catalyst material (Scheme 1).

**FIGURE 1:**
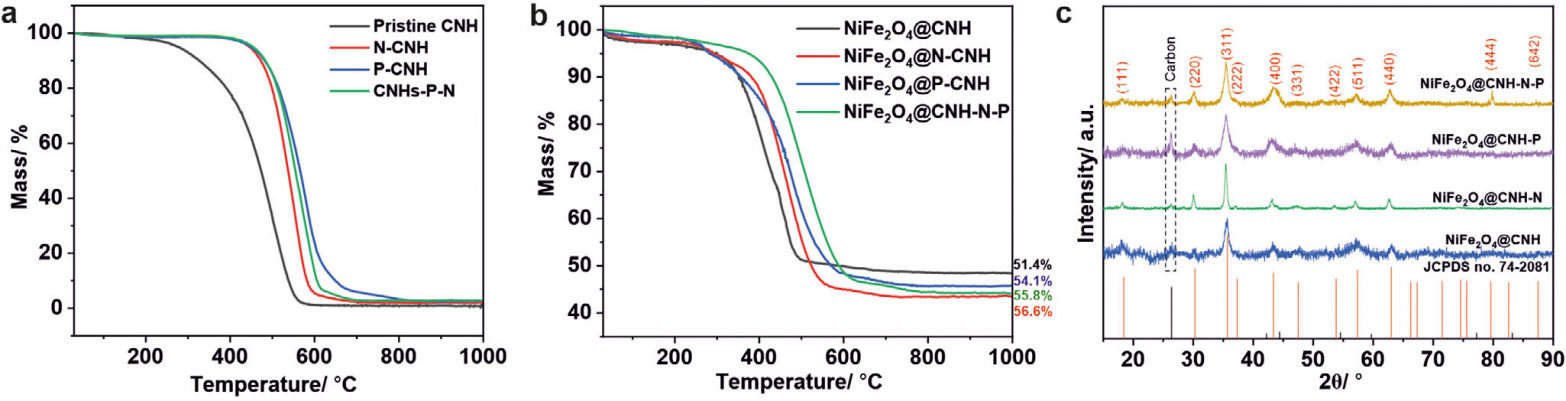
Thermogravimetric analysis of (a) different types of CNHs, (b) different types of nanohybrids, and (c) XRD of different nanohybrids.

The synthesis comprises a two‐step process (Scheme 1). First, CNH is functionalized (Step I), followed by the synthesis of the nanohybrid (Step II). The functionalization step resulted in four types of carbon nanohorns: pristine CNH, N‐CNH, P‐CNH, and CNH‐N‐P. Using each type of carbon nanohorn, four nanohybrids were synthesized: NiFe_2_O_4_@CNH, NiFe_2_O_4_@N‐CNH, NiFe_2_O_4_@P‐CNH, and NiFe_2_O_4_@CNH‐N‐P (Table S1). Figure 1a shows thermogravimetric analysis of different CNHs and corresponding nanohybrids. Pristine CNH exhibits one‐step total mass loss, initiating at ∼217 – 582°C, corresponding to oxidative decomposition of the carbon nanohorns framework. After heteroatom incorporation and heat treatment, N‐CNH, P‐CNH, and CNH‐N‐P exhibit a shift of the decomposition temperature from ∼385 up to ∼484°C, indicating increased thermal stability [26]. The low residual mass (<5%) observed across all samples confirms their purity with minimal inorganic impurities. The improved oxidation resistance of the doped CNHs is remarkable and suggests enhanced structural strength, although heteroatom doping may also increase defect density. Both situations should promote electrocatalytic stability in oxygen electrochemistry [23]. In the TG analysis, all nanohybrid samples exhibit a gradual mass loss below ∼300°C, attributed to physically adsorbed water and surface functional groups (Figure 1b). An additional significant mass loss between 350°C and 590°C is attributed to a degradation of the CNH carbon framework. Nevertheless, a remarkable shift in the degradation temperature of the heteroatom (N, P)‐doped carbon nanohorn‐hybrid indicates improved thermal stability of the carbon support after heteroatom incorporation. The corresponding residual mass can be attributed to the thermostable phase of NiFe_2_O_4_, thereby enabling estimation of the relative metal oxide loading in the nanohybrids. The enhanced thermal stability and higher residual mass observed for NiFe_2_O_4_@CNH‐N‐P may reflect the stronger interfacial interaction between NiFe_2_O_4_ nanoparticles and N, P‐doped CNH framework, as well as a high active site density. Both together facilitate charge transfer and oxygen adsorption, thereby improving bifunctional catalytic activity [7, 27].

X‐ray diffraction patterns determine and confirm the crystal structure and phase purity of all samples. Pure NiFe_2_O_4_ nanoparticles (Figure S2a) and the nanohybrids (Figure 1c) synthesized by the hydrothermal method exhibit reflections corresponding to characteristic peaks of the spinel phase of NiFe_2_O_4_ (PDF#74‐2081). In the nanohybrids, an additional peak at 26° (2θ) corresponds to the graphitic carbon peak. The average crystallite size of the synthesized samples was estimated using the Debye–Scherrer equation based on the five most intense diffraction peaks. The crystallite size of NiFe_2_O_4_ nanoparticles is ∼18.5 nm, and in the nanohybrids it ranges from 7 to 8.5 nm (Table S1). The crystallite size of NiFe_2_O_4_ in the hybrids is relatively small owing to the presence of CNH, which acts as a matrix/structure to provide support for the small and uniform development of the metal oxide nanoparticles [14]. As such, it may allow early anchoring on the CNH‐N‐P surface compared to the bare ferrite formation process, thereby suppressing further particle growth by blocking active growth sites on the particle surface.

**FIGURE 2:**
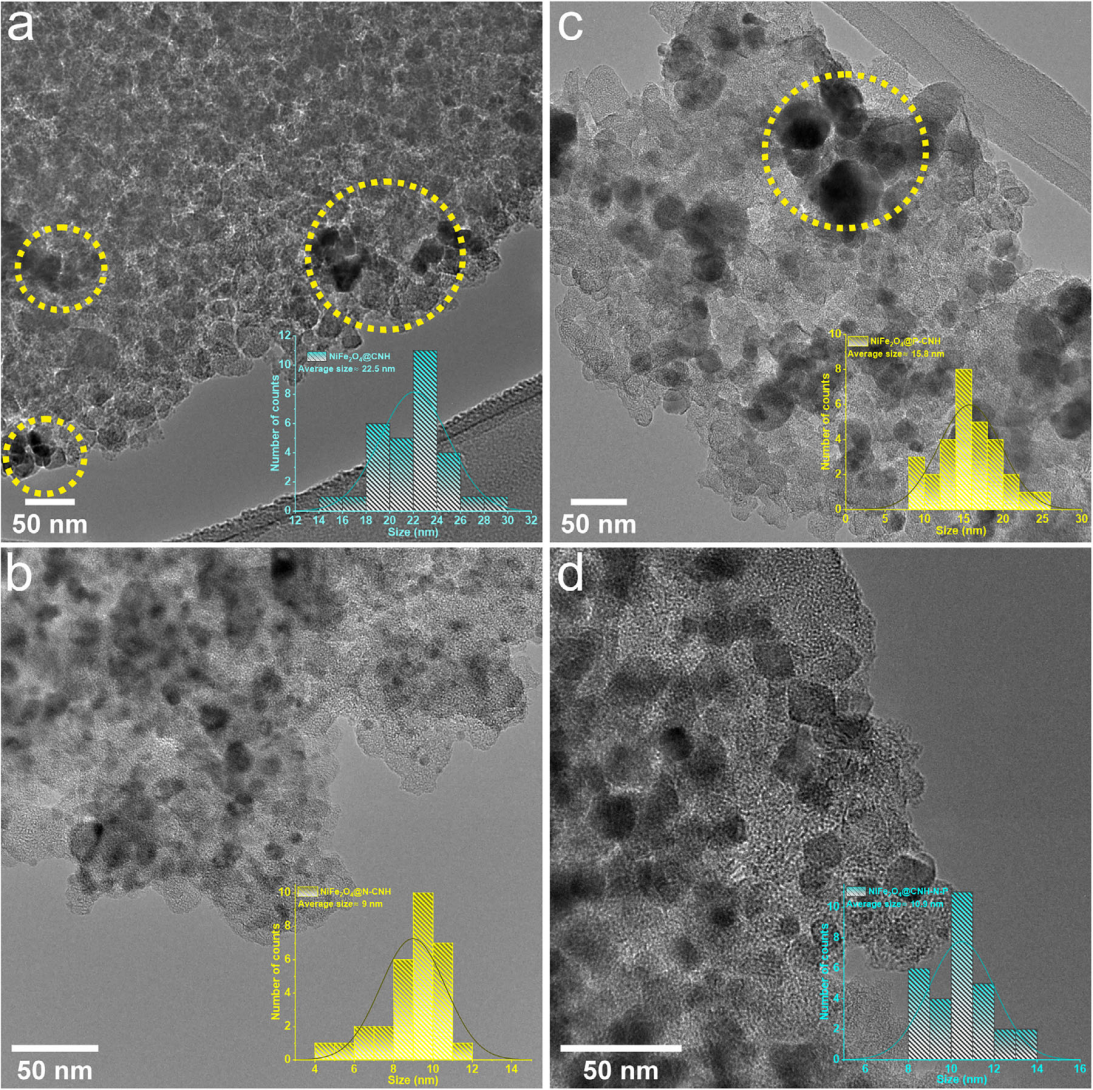
TEM images of nanohybrids, (a) NiFe_2_O_4_@CNH, (b) NiFe_2_O_4_@N‐CNH, (c) NiFe_2_O_4_@P‐CNH, and (d) NiFe_2_O_4_@CNH‐N‐P.

Furthermore, the structure and morphology of NiFe_2_O_4_, CNH, functionalized CNH, and the NiFe_2_O_4_@CNH‐hybrid are characterized using transmission electron microscopy (TEM). TEM image of pure NiFe_2_O_4_ (Figure S2b) shows aggregated polygonal nanoparticles with predominantly 50–100 nm in size, yielding an average particle diameter of 84.3 nm. Many particles exhibit irregular faceted morphology, with some small particles having a cubic structure (encircled in red). High‐resolution transmission electron micrographs (HRTEM) of NiFe_2_O_4_ exhibit lattice fringes with interplanar distances of 0.24, 0.25, and 0.48 nm, characteristic of the (222), (311), and (111) planes of its spinel phase NiFe_2_O_4_ (JCPDS No. 74–2081) (Figure S2c). Similarly, the SAED pattern of NiFe_2_O_4_ NPs shows bright spots forming diffraction rings, which correspond to the polycrystalline nature of the spinel NiFe_2_O_4_ nanoparticles assigned to (311), (222), (422), (444), (440), and (533) crystallographic planes (Figure S2d). The pristine CNH displays bud‐like morphology (Figure S3a) with a diameter of ∼50 nm. After N‐functionalization, there is no significant change in the morphology; however, at several spots, nanohorns exhibit more distinct openings (Figure S3b). Similarly, P‐functionalized and N, P‐functionalized CNH do not show substantial variation at the morphological level; however, the end opening can be clearly seen and confirmed by protruding single cones at several spots (green circular spots shown in Figure S3c,d). The NiFe_2_O_4_@CNH nanohybrid electron micrograph shows a nonuniform distribution of NiFe_2_O_4_ nanoparticles within the pristine CNH matrix with an average particle size of ∼22.5 nm (Figure 2a). In contrast, the electron micrographs of NiFe_2_O_4_@N‐CNH and NiFe_2_O_4_@P‐CNH (Figure 2b,c) show a uniform distribution, though with still a few aggregates left (highlighted in yellow circles) displaying an average particle size of 9.5 and 15.8 nm, respectively. The NiFe_2_O_4_@CNH‐N‐P nanohybrid electron micrograph (Figure 2d) shows a uniform distribution of NiFe_2_O_4_ nanoparticles within the CNH‐N‐P matrix. The NiFe_2_O_4_ in NiFe_2_O_4_@CNH‐N‐P exhibits uniform cubic morphology with an average size of ∼10.4 nm.

**FIGURE 3:**
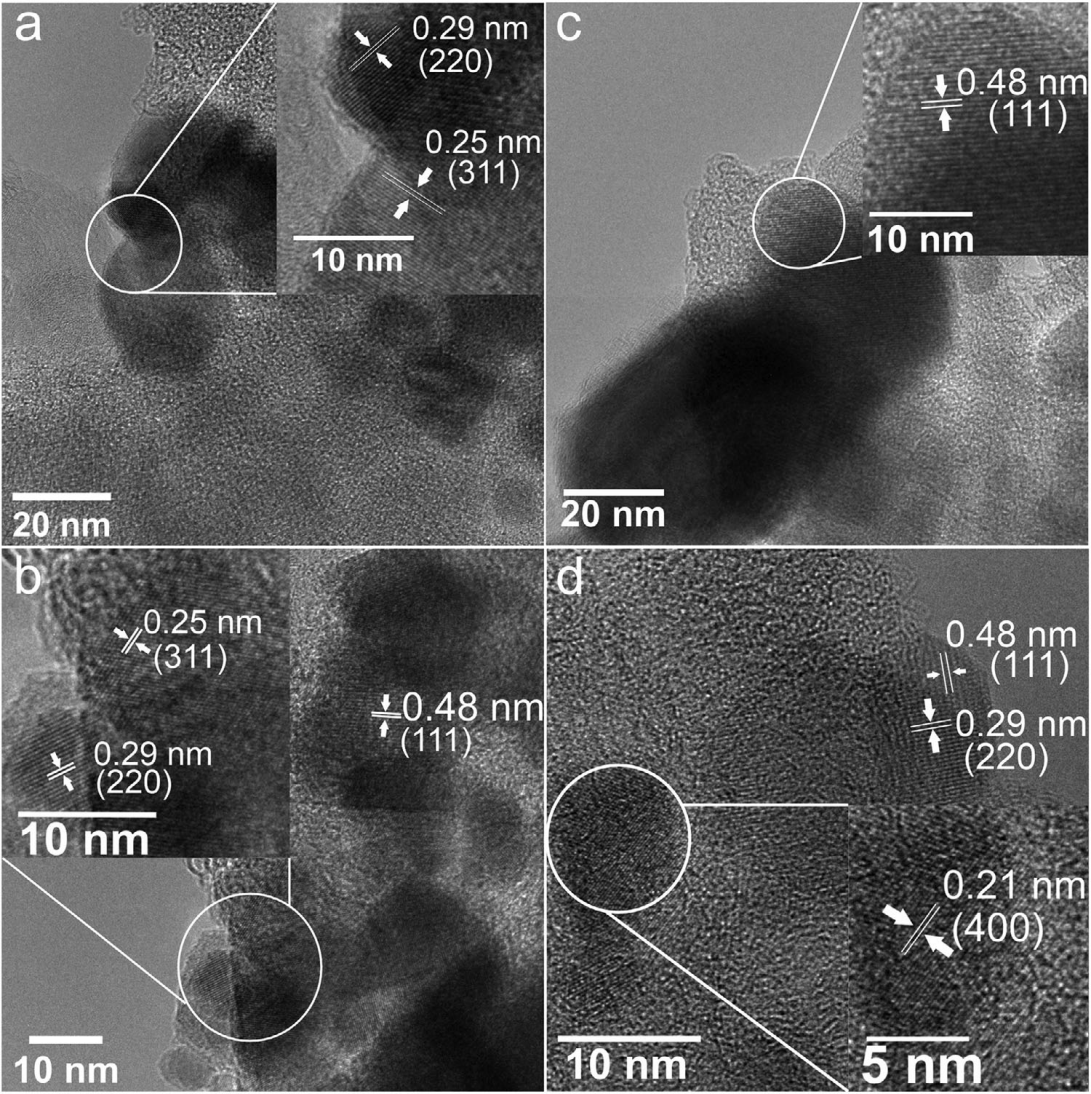
HRTEM images of nanohybrids, (a) NiFe_2_O_4_@CNH, (b) NiFe_2_O_4_@N‐CNH, (c) NiFe_2_O_4_@P‐CNH, and (d) NiFe_2_O_4_@CNH‐N‐P.

TEM analysis is also helpful for confirming the phase purity and structure of crystalline materials. HR‐TEM of all nanohybrids is shown in Figure 3. HRTEM of nanohybrids exhibits lattice fringes with d‐spacings characteristic of nickel ferrite (PDF#74‐2081). The evident d‐spacings are 0.48, 0.29, 0.25, and 0.20 nm, corresponding to planes (111), (220), (311), (222), and (400), respectively. HRTEM of NiFe_2_O_4_@CNH‐N‐P nanohybrid (Figure 3d) clearly shows the adherence of NiFe_2_O_4_ cubes over the CNH‐N‐P matrix with an intimate contact boundary between the lattice fringes of NiFe_2_O_4_ and the graphitic layers of CNH‐N‐P, confirming the interfacial coupling [28]. The SAED patterns of the nanohybrids (Figure S4) show bright spots forming diffraction rings, which confirms the polycrystalline nature of the NiFe_2_O_4_ component in the nanohybrids. The rings are assigned to (111), (220), (311), (222), (400), (422), (331), (442), (622), and (642) planes of the spinel phase NiFe_2_O_4_.

Raman spectroscopy helps assess the crystal integrity of the carbon materials. It provides convincing evidence of the superficial defects created and the presence of heteroatom dopants in the CNH structure. Pristine CNH exhibits two broad peaks, 1327 and 1581 cm^−1^, corresponding to the characteristic D and G bands of carbon, respectively (Figure S5a). The I_D_/I_G_ for the functionalized CNH is higher relative to pristine CNH and in the sequence CNH (1.64) < N‐CNH (1.80) < P‐CNH (1.91) < CNH‐N‐P (1.99) (Table S2). Similarly, nanohybrids prepared with functionalized CNH exhibit higher I_D_/I_G_ ratios than those prepared with pristine CNH. These findings indicate that functionalized CNHs exhibit the maximum degree of disorder due to surface defects introduced by the functionalization/hetero‐element doping route. During functionalization with urea (N) and phytic acid (P), high‐temperature treatment enhances both etching and heteroatom incorporation in CNH. The induced defect spots, along with the N and P heteroatoms, serve as active centres for oxygen reduction. Functionalization of CNH is additionally proven by a change in the peak position of the G band and 2D band to a higher wavenumber in N‐CNH, P‐CNH, and CNH‐N‐P and the nanohybrids (Table S2). The electron‐retreating influence of the heteroatom dopants induces a redshift in the G‐band and 2D band positions of the carbon network [15, 26, 29, 30]. Raman spectra of the NiFe_2_O_4_@CNH‐N‐P nanohybrids exhibit additional five signals at lower energies: 284, 371, 474, 575, and 690 cm^−1^ corresponding to characteristic phonon modes of the tethered spinel nickel ferrite. The peak at 371 cm^−1^ is attributed to the E_g_ vibrational modes, peaks at 284, 474, and 574 cm^−1^ are attributed to the T_2g_ vibrational mode, and the peak at 690 cm^−1^ is attributed to A_1g_ vibrational modes [31] (Figure S5b).

The representations A, E, and T denote one, two, and three dimensions of phonon modes, and g denotes the centre of inversion symmetry. Along Ni─O and Fe─O bonds, the symmetric stretching of oxygen atoms at tetrahedral sites is assigned to the A_1g_ mode. In addition, symmetric bending of oxygen with reference to the Fe and Ni ions assigned to the E_g_ mode, and T_2g_ (574 cm^−1^) mode signifies the asymmetric bending of the oxygen bond. The T_2g_ (475 cm^−1^) mode is assigned to the asymmetric stretching of metal ions and O. The mode T_2g_ (284 cm^−1^) is assigned to the translational motions of the tetrahedron (tetrahedral site metal atoms along with oxygen atoms) [32]. Raman studies thus confirm the formation of the efficient nanohybrid structure by the two‐step method (Scheme 1). First, CNH is pre‐functionalized, followed by hydrothermal synthesis and subsequent tethering of NiFe_2_O_4_ nanoparticles in the presence of CNH, either in its native form or functionalized.

**FIGURE 4:**
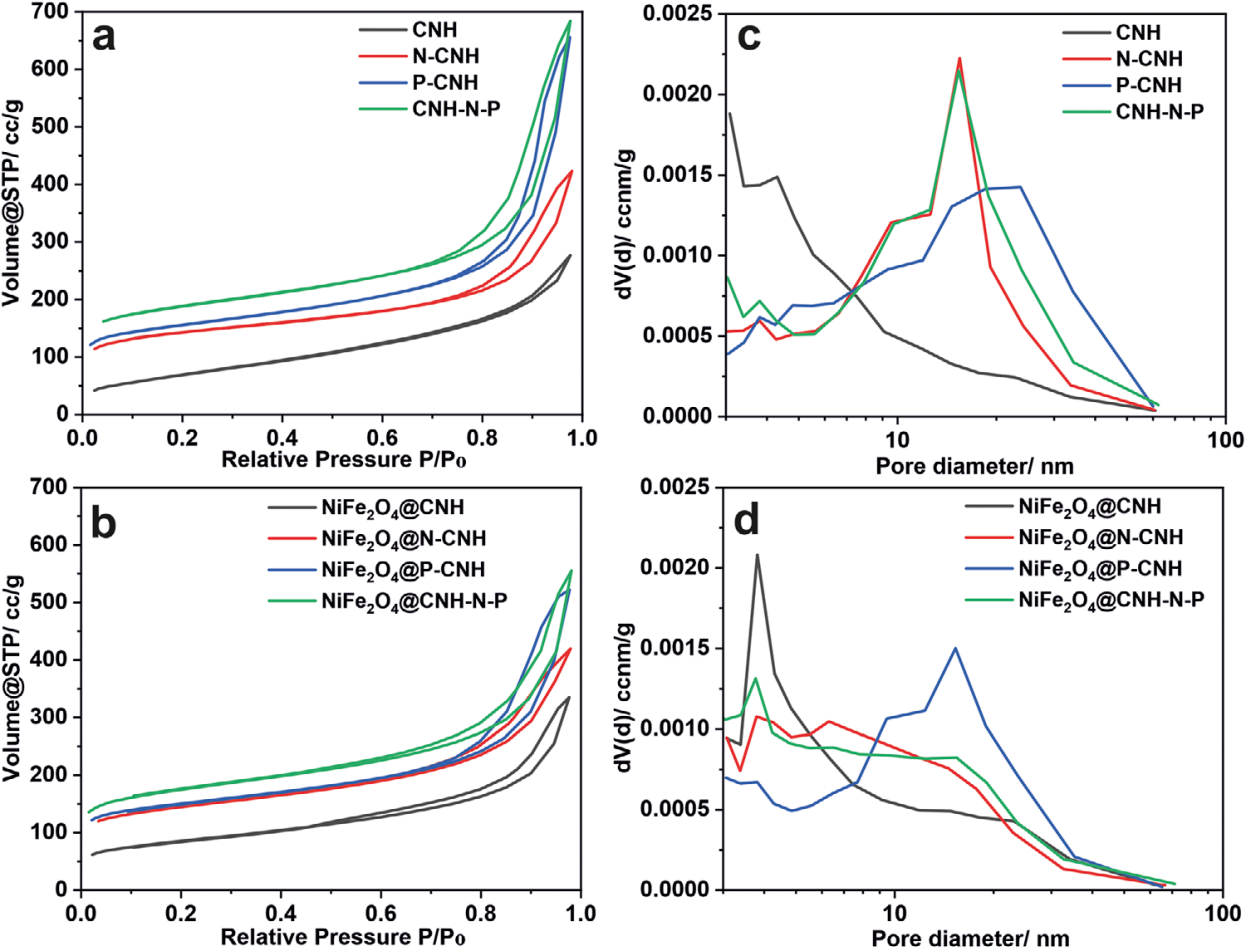
(a, b) Nitrogen adsorption‐desorption isotherms and (c, d) BJH pore size distribution of pristine CNH, N‐CNH, P‐CNH, CNH‐N‐P, and the nanohybrids NiFe_2_O_4_@CNH, NiFe_2_O_4_@N‐CNH, NiFe_2_O_4_@P‐CNH, and NiFe_2_O_4_@CNH‐N‐P.

The porosity of all CNHs and NiFe_2_O_4_@CNH, NiFe_2_O_4_@N‐CNH, NiFe_2_O_4_@P‐CNH, and NiFe_2_O_4_@CNH‐N‐P nanohybrids was characterized using surface analysis. The specific surface areas of the samples were determined using the Brunauer‐Emmett‐Teller (BET) theory. The pristine CNH exhibits a surface area of 258.4 m^2^/g. After functionalization, the values increase to 529.3 m^2^/g for N‐CNH, 576.6 m^2^/g for P‐CNH, and 695.9 m^2^/g for CNH‐N‐P. CNH‐N‐P reveals a nearly threefold increase in the surface area relative to the pristine CNH due to heteroatom (dual) functionalization. Among the nanohybrids, NiFe_2_O_4_@CNH‐N‐P exhibits a higher surface area of 625 m^2^/g (all surface analysis characteristics are listed in Table S3). The N_2_ adsorption‐desorption isotherms and BJH pore size distribution curves of pristine CNH and modified CNHs, along with their corresponding NiFe_2_O_4_ nanohybrids, are shown in Figure 4a–d. All samples (CNHs and nanohybrids) exhibited type IV isotherms, indicating the presence of consistent mesoporous structures with a narrow size distribution. The gradual adsorption at low relative pressure (P/P_0_<0.1) suggests the presence of micropores [33, 34], while the continuous increase in adsorption at intermediate pressure is attributed to multilayer adsorption and mesopore filling. A sharp rise in adsorption capacity at high relative pressure (P/P_0_>0.8) suggests the presence of mesoporous space, which can be attributed to the interstitial space between aggregated nanohorns. Compared to pristine CNH, the functionalized CNHs exhibit higher adsorption capacities. This improvement is due to additional porosity, attributed to structural modifications such as the removal of amorphous carbon, opening of nanohorn tips, and partial etching of the graphitic wall structure of the CNH. Among the functionalized samples, CNH‐N‐P exhibits the highest adsorption capacity, indicating the formation of more developed porous architecture after dual modification. The nanohybrids maintain mesoporosity throughout; however, a slight decrease in adsorption capacity is observed compared with bare CNH supports. This decrease is due to partial pore blocking caused by the tethering of the nickel ferrite nanoparticles. It reduces accessible pore volume and surface area. Notably, among the nanohybrids, NiFe_2_O_4_@CNH‐N‐P exhibits the highest adsorption capacity, indicating that the porous structure generated by dual modification is stable, effectively accommodates ferrite nanoparticles, and maintaining a relatively large accessible surface area.

An inspection of the pore size distribution (Figure 4c) further confirms the evolution of porous structures after functionalization of CNH. Pristine CNH primarily exhibits small mesopores in the of 3–5 nm range. After modification (functionalization), the porosity type of CNH shifts toward larger mesopores (∼10‐15 nm) and shows an increase in pore volume (Table S3). For the nanohybrids (Figure 4d), the pore size distribution remains within the mesoporous region, but the pore volumes are slightly reduced compared to corresponding CNH supports (Table S3).

The porous structure observed in functionalized CNH should be highly beneficial for electrochemical reactions (ORR/OER). The high surface area provides abundant exposed sites, while mesopores facilitate rapid electrolyte diffusion and efficient mass transport. Because of its high surface area, high pore volume, and increased micropore area, NiFe_2_O_4_@CNH‐N‐P is expected to exhibit an enhanced ORR‐OER activity [15, 33].

**FIGURE 5:**
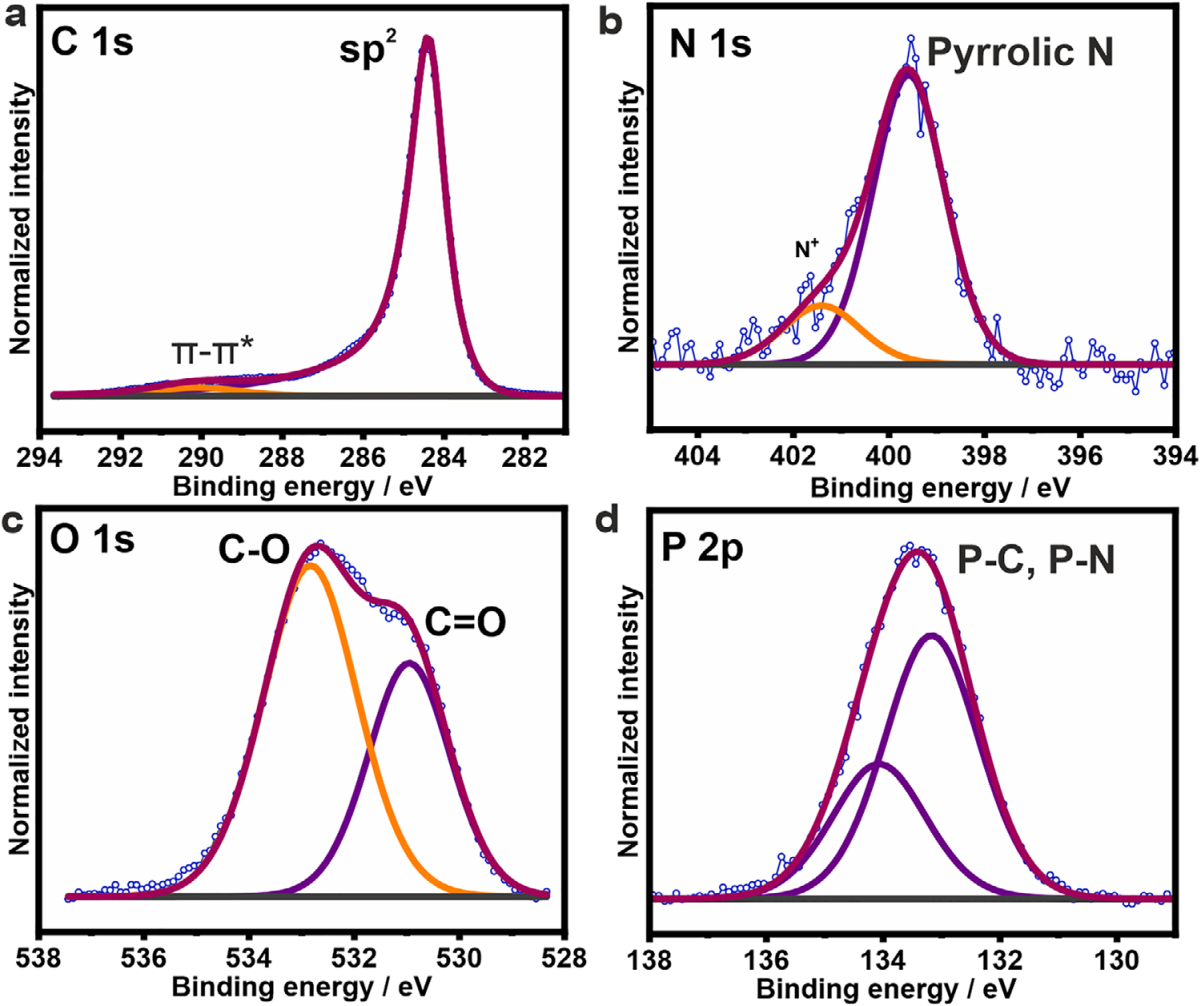
(a) High‐resolution spectra of CNH‐N‐P, (a) C 1s, (b) N 1s, (c) O 1s, and (d) P 2p.

Chemical surface information of pristine CNH and functionalized CNHs was obtained from XPS/high resolution XPS. Pristine CNH exhibits C 1s, N 1s, O 1s, C KLL, and O KLL signals (Figure S6a). Annealing of the CNH with urea and phytic acid results in nitrogen and phosphorus functionalization, and considerably lessens the oxygen content (Table S4), confirmed by a drop in total oxygen content (13.2 to 3.8 at.%) and evident in high‐energy‐resolution C 1s spectra depicting a decrease in *π*–*π* ^*^ signal intensity (Figure S6b, which shows comparative C 1s of CNH, N‐CNH, and CNH‐N‐P) [35]. Survey spectra of CNH‐N‐P (Figure S6a) indicate the presence of C, N, O, and P without any other contaminants. The presence of N and P in the survey spectrum of CNH‐N‐P confirms its doping with nitrogen and phosphorus. The high‐resolution C 1s spectrum reveals (Figure 5a) two peaks at 284.4 and 289.9 eV corresponding to sp^2^ carbon and the *π*–*π* ^*^ shake‐up characteristic signals of graphitic features present in the CNH, respectively. Compared to pristine CNH, the C 1s peak of CNH‐N‐P exhibits slight broadening and asymmetry, depicting enhanced structural disorder and heteroatom incorporation. O 1s spectra in all CNHs exhibit slight contributions from carboxyl groups, C═O (530–531 eV) and C─O (532–533 eV), respectively (Figure S6d) [26, 36].

To understand electrocatalytic activity, a detailed discussion on N and P doping is required (Figure 5b,d). The high‐resolution N 1s spectrum after N functionalization (N‐CNH) consists of three peaks at 398.5, 400.7, and 403.6 eV, corresponding to pyridinic N, pyrrolic N, and nitrogen oxide (NOx), respectively (Figure S6c, shows comparative N 1s of CNH, N‐CNH, and CNH‐N‐P). However, in CNH‐N‐P, N 1s consists of only two dominant peaks at 399.6 and 401.0 eV, attributed to pyrrolic N and quaternary N, respectively. This change is attributed to the second step, the P functionalization, which includes an additional heating step, resulting in a decrease of pyridinic N and NO_x_ species and an increased proportion of pyrrolic/quaternary nitrogen [37]. CNH‐N‐P exhibits a percentage of 2.3 at.% phosphorus doping. The high‐resolution P 2p doublet with P 2p_3/2_ at 133.2 eV binding energy corresponds to phosphorus bound either to C or to N (Figure 5d and Figure S6e) [38]. Oxidized phosphorus would appear at a higher binding energy. Remarkably, the P signal intensity (atomic %) is significantly higher than P‐CNH, consistent with P anchoring/functionalization following the prior N‐functionalization [3, 16].

**FIGURE 6:**
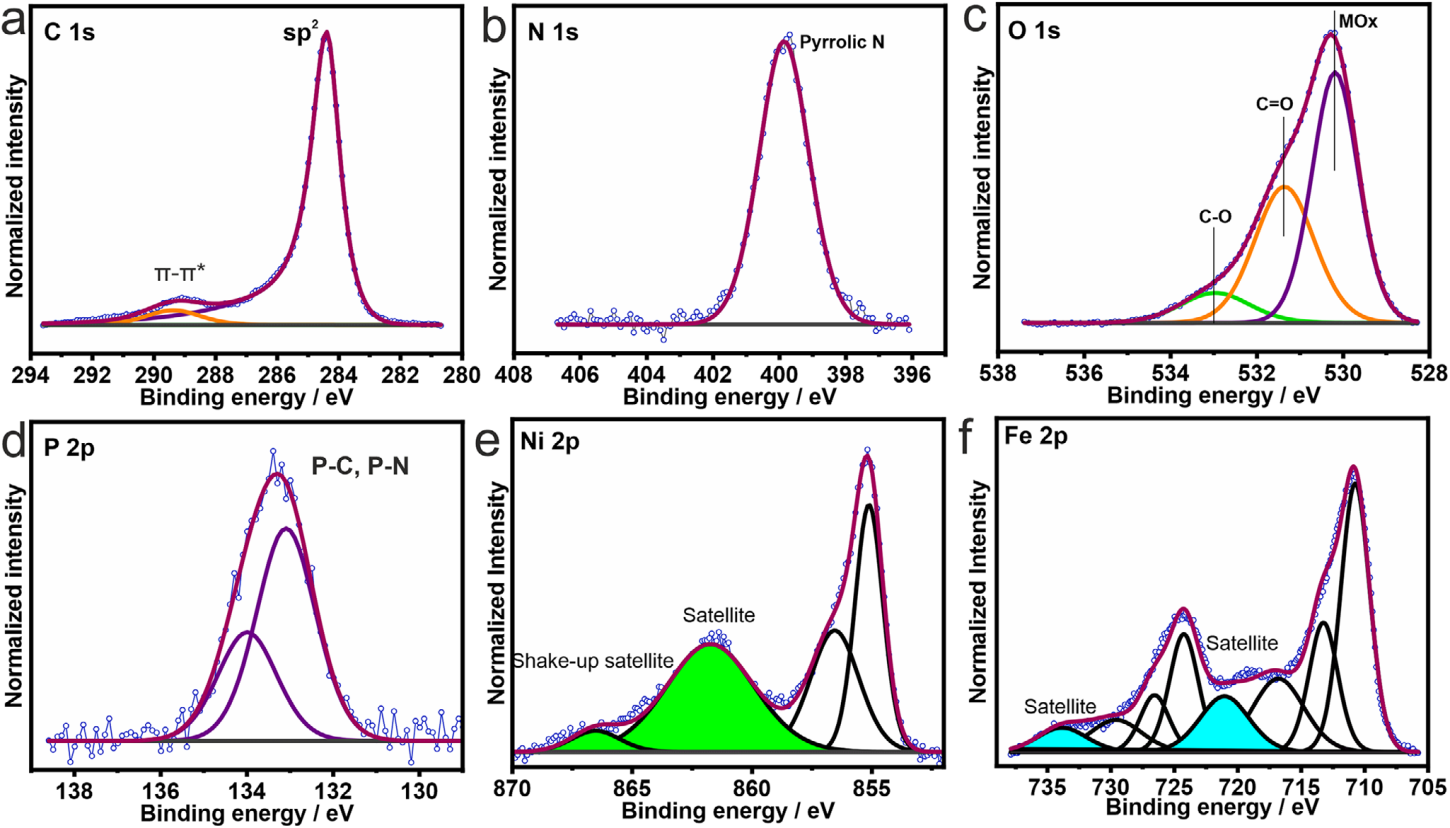
(a) High‐resolution XPS spectra of the nanohybrid NiFe_2_O_4_@CNH‐N‐P, showing (a) C 1s, (b) N 1s, (c) O 1s, (d) P 2p, (e) Ni 2p, and (f) Fe 2p spectra.

Subsequently, the surface chemical composition and electronic states of NiFe_2_O_4_@CNH, NiFe_2_O_4_@N‐CNH, NiFe_2_O_4_@P‐CNH, and NiFe_2_O_4_@CNH‐N‐P nanohybrids were also analysed using XPS/high‐resolution XPS. The survey spectra of nanohybrids NiFe_2_CO_4_@CNH and NiFe_2_O_4_@N‐CNH exhibit characteristic peaks for C, N, O, Ni, and Fe elements (Figure S7). Nanohybrids NiFe_2_O_4_@P‐CNH and NiFe_2_O_4_@CNH‐N‐P exhibit characteristic peaks for P, C, N, O, Ni, and Fe elements (Figure S7). The survey spectrum of NiFe_2_O_4_@CNH‐N‐P confirms the presence of C, N, O, P, Ni, and Fe elements in the functionalized nanohybrids, indicating successful incorporation of heteroatoms and metal oxide onto the CNH framework (Figure S7). The high‐resolution XPS spectra of C 1s, N 1s, O 1s, P 2p, Ni 2p, and Fe 2p of the NiFe_2_O_4_@CNH‐N‐P nanohybrid are shown in Figure 6. The C 1s and N 1s spectra retain features of CNH‐N‐P, indicating preservation of the conductive carbon matrix [39]. The O 1s spectrum shows enhanced contribution from lattice oxygen (MOx) at 530.0 eV, consistent with the presence of NiFe_2_O_4_, along with surface oxygen species associated with the carbon framework. The high‐resolution Ni 2p spectrum exhibits typical Ni 2p_3/2_ peaks at 855.1, 856.6 eV, characteristic of Ni^2+^, along with satellite peaks at 861.8 eV and a shake‐up satellite peak at 866.6 eV [7, 40]. Fe 2p spectrum shows multiple peak fits consistent with predominant Fe^3+^ (satellite at ∼720 eV) with possibly minor Fe^2+^ contributions [15]. The elemental composition ratio obtained from quantitative analysis of XPS data indicates the formation of the NiFe_2_O_4_ phase. The P 2p_3/2_ doublet peak of phosphorus remains around 133 eV, confirming that phosphorus incorporation in the CNH framework is stable upon oxide deposition. The peaks are attributed to phosphorus bound to either C or N [38]. Upon NiFe_2_O_4_ integration, there is a substantial increase in the atomic percentage of oxygen and nitrogen species in the nanohybrids (Table S4). The increasing trends in nitrogen and oxygen are attributed to urea complex decomposition and the ferrite formation step, as the urea complex decomposition induces further N functionalization of the CNH [14, 22]. Overall, the XPS results confirm codoping of CNH‐N‐P (N = 2.2 at.%, and p = 2.3 at.%) and NiFe_2_O_4_@CNH‐N‐P (N = 4.1 at.%, and p = 1.6 at.%). N, P doping will lead to efficient charge transfer to the carbon framework and will eventually amplify the catalytic activity of the nanohybrids [3, 41]. Furthermore, compared to pure NiFe_2_O_4_ (Table S5) the Fe 2p_3/2_ and Ni 2p_3/2_ core levels both shift to higher binding energy by 0.2 ∼0.3 eV in the composite relative to pure NiFe_2_O_4_, and the N 1s core level shifts to higher binding energy by 0.3 eV in the composite relative to only CNH‐N‐P. These correlated binding‐energy shifts across independent metal (Fe, Ni) and dopant (N) core levels indicate a redistribution of electron density at the NiFe_2_O_4_/CNH‐N‐P interface upon hybrid formation, consistent with genuine interfacial coupling rather than the simple co‐presence of unmodified components [42, 43, 44].

**FIGURE 7:**
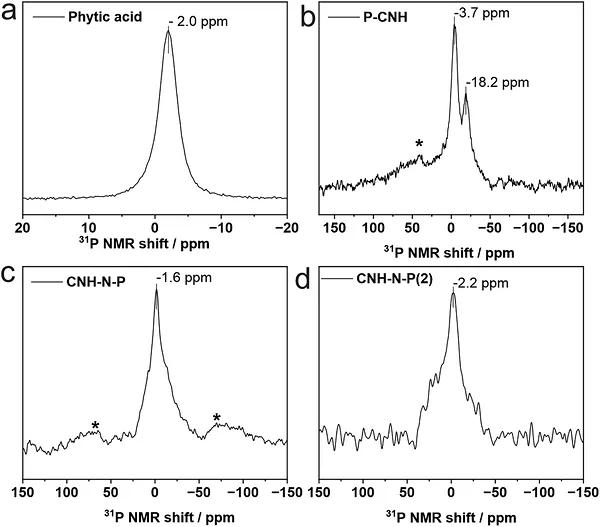
^31^P NMR spectrum of (a) phytic acid and ^31^P MAS NMR spectra of (b) P‐CNH, (c) CNH‐N‐P, and (d) CNH‐N‐P(2), measured at 7 T. Main peaks are marked with their corresponding chemical shifts. The number of scans was varied from 8 (a) to 8129 (b), 1024 (c), and 4000 scans (d) depending on spectrum resolution, signal‐to‐noise ratio, and available measurement time. Liquid phytic acid was measured under static conditions (a) while the MAS frequency for solid samples was varied from 5 kHz for (b) to 8 kHz for (c), and 6.25 kHz for (d) depending on rotor stability and resolution. Asterisks (^*^) denote spinning sidebands due to MAS.

To inspect the chemical environment and bonding arrangement of phosphorus in functionalized CNH, ^31^P solid‐state nuclear magnetic resonance (ssNMR) spectroscopy using magic angle spinning (MAS) was performed. The ^31^P ssNMR spectrum of phytic acid (Figure 7a) precursor exhibits a sharp resonance at −2.0 ppm, consistent with six equivalent phosphate groups of the inositol hexaphosphate structure [45, 46, 47]. The ^31^P MAS NMR of P‐CNH (Figure 7b) exhibits two primary resonances at −3.7 ppm and −18.2 ppm, attributed to surface‐anchored phosphate species and condensed pyrophosphate groups, respectively [48].

In the co‐doped CNH‐N‐P material (Figure 7c), the ^31^P surrounding undertakes a remarkable structural simplification. The spectra are dominated by a primary resonance shifting slightly downfield to −1.6 ppm. This transition suggests that the introduction of nitrogen acts as a structural stabilizer, promoting a more uniform distribution of phosphorus species in the CNH‐N‐P as compared to the phytic acid‐treated CNH. The broadening of the signals in the CNH‐N‐P samples indicates a high degree of structural disorder and numerous lattice defects [38]. This suggests a synergistic effect between the incorporated elements phosphorous and nitrogen.

SEM‐EDX analysis is an important technique for determining morphology and surface uniformity at the upper‐nm scale. The SEM image of CNH‐N‐P (Figure S8a) reveals an entangled, porous nanohorn network with a coarse surface morphology, attributable to functionalization steps and beneficial for exposing active sites and facilitating mass transport. The corresponding EDX elemental mapping (Figure S8b) confirms the uniform distribution of C, O, N, and P throughout the carbon framework, indicating successful heteroatom incorporation without phase segregation. The homogeneous dispersion of N and P dopants induces charge redistribution within the carbon lattice, thereby enhancing electrical conductivity and catalytic activity. For the NiFe_2_O_4_@CNH‐N‐P nanohybrid, the SEM image shows well‐dispersed nanoparticles anchored to the CNH‐N‐P matrix (Figure S9a), with no noticeable agglomeration. Elemental mapping (Figure S9b) demonstrates the uniform distribution of Ni and Fe, along with C, N, O, and P, confirming the successful integration of NiFe_2_O_4_ within the doped carbon framework and ensuring strong interfacial coupling between the metal oxide and support, which is critical for efficient electron transfer and enhanced catalytic performance.

Thus, the combined N and P doping, along with interfacial coupling between CNH and NiFe_2_O_4_ (exhibited by TEM, HRTEM, and XPS analysis), could lead to further enhanced electronic properties in the nanohybrid NiFe_2_O_4_@CNH‐N‐P. This synergistic effect could enhance charge transfer, increase active‐site density, and improve bifunctional catalytic performance.

### 2.2 Studies on the Electrochemical Activity (ORR and OER) of CNHs and NiFe_2_O_4_ Tethered on CNHs

**FIGURE 8:**
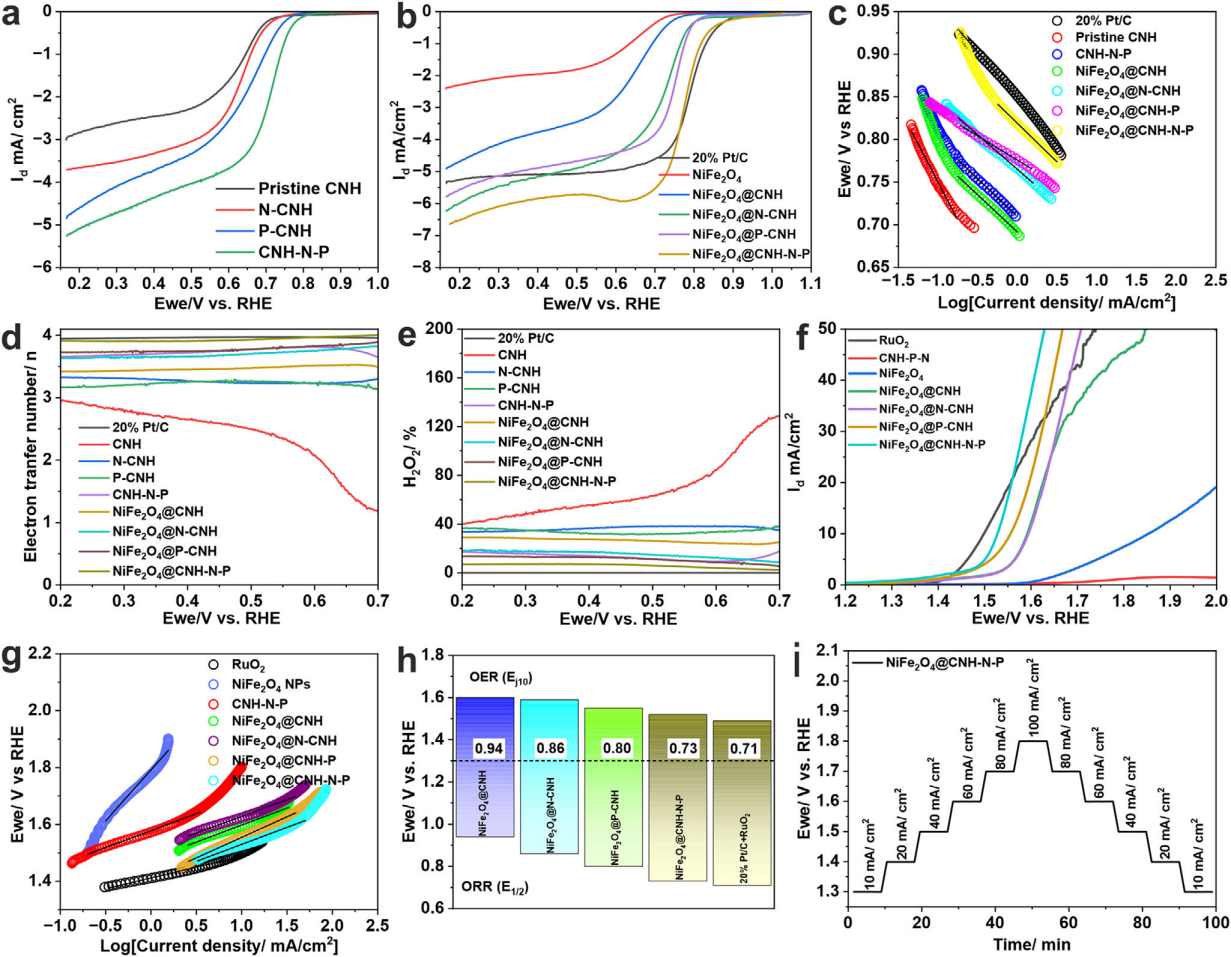
(a) ORR polarization curves of CNH, N‐CNH, P‐CNH, and CNH‐N‐P, (b) ORR polarization curves of NiFe_2_O_4_@CNH, NiFe_2_O_4_@N‐CNH, NiFe_2_O_4_@P‐CNH, and NiFe_2_O_4_@CNH‐N‐P nanohybrids compared to 20% Pt/C. (c) Tafel plots of ORR measurements. From RRDE measurements determined, (d) number of electrons transferred (n), and (e) percentage of peroxide formation (H_2_O_2_). (f) OER polarization curves for all the samples, (d) Tafel plots of OER measurements. (h) potential difference between E_1/2_ and OER potential at 10 mA/cm^2^ of different nanohybrids. Chronopotentiometry measurements of NiFe_2_O_4_@CNH‐N‐P at different current densities. All measurements (ORR‐OER) were performed in 0.1 m KOH.

To determine the electrochemical activity of the modified CNHs and synthesized nanohybrids therefrom, RDE and RRDE measurements were performed in O_2_‐saturated 0.1 m KOH. Figure 8a,b show the ORR activity of all prepared samples systematically evaluated using linear sweep voltammetry (LSV) at 1600 rpm (for LSV plots at different rotation rates, 400–2500 rpm, see Figures S10 and S11). Pristine CNH exhibits a low half‐wave potential of E_1/2_ = 0.63 V vs. RHE and a low limiting current density of −3.0 mA/ cm^2^. After heteroatom doping, the activity improves significantly, with N‐CNH and P‐CNH showing E_1/2_ values of 0.68 and 0.69 V vs. RHE, respectively (Table S6). In comparison, co‐doped CNH‐N‐P reaches E_1/2_ = 0.71 V vs. RHE with an enhanced limiting current density of −5.2 mA/ cm^2^. The superior ORR performance of the CNH‐N‐P catalyst compared with N‐CNH and P‐CNH can be attributed to the synergistic effect of dual N and P doping, rather than to the individual contributions of either heteroatom. Nitrogen doping introduces catalytically active sites and improves the electronic conductivity of the carbon framework, whereas phosphorus, owing to its larger atomic radius and lower electronegativity, induces lattice distortion, generates additional defects, and modifies asymmetric spin and charge distribution of adjacent carbon atoms, thereby promoting oxygen adsorption and charge transfer [16, 17, 41, 49, 50]. This synergistic effect is further supported by the XPS results. After the second heat treatment during P functionalization, the nitrogen environment changes from pyridinic N, pyrrolic N, and NOx species in N‐CNH to predominantly pyrrolic and quaternary nitrogen in CNH‐N‐P, accompanied by the disappearance of NOx species. Moreover, the phosphorus content increases significantly from 0.5 at.% in P‐CNH to 2.3 at.% in CNH‐N‐P, indicating more effective phosphorus incorporation after nitrogen functionalization [51]. Consistent with this mechanism, the co‐doped CNH‐N‐P exhibits the highest defect density (I_D_/I_G_ = 1.99), the largest BET surface area (695.9 m^2^/g), and the highest micropore area among all samples, thereby providing more accessible electrochemically active sites. Furthermore, the improved ORR selectivity is evidenced by an increased electron‐transfer number (3.69) and a significantly reduced H_2_O_2_ yield (17.2%) compared with N‐CNH and P‐CNH. These results indicate that the enhanced activity of CNH‐N‐P originates from the combined electronic and structural effects of N and P co‐doping, rather than a simple additive effect of the two individual dopants. A further noteworthy improvement is observed in nanohybrids, as shown in Figure 8b. Among all the nanohybrid catalysts, NiFe_2_O_4_@CNH‐N‐P exhibits the highest E_1/2_ of 0.79 (Table S6), matching the commercial ORR catalyst 20% Pt/C (0.80 V), with the largest limiting current density of ‐6.6 mA/cm^2^. The Tafel slope of the NiFe_2_O_4_@CNH‐N‐P nanohybrid is significantly reduced to 56.5 mV/dec, compared to pristine (178 mV/dec) and 80–95 mV/dec for other nanohybrids, indicating accelerated reaction kinetics (Figure 8c). These results highlight the strong synergistic interfacial coupling between NiFe_2_O_4_ and N, P co‐doped CNH support, which facilitates rapid charge transfer and optimizes the adsorption of oxygen intermediates [52, 53, 54]. Figure 8d,e further elucidate the ORR mechanism, as revealed by RRDE measurements (Table S7). The NiFe_2_O_4_@CNH‐N‐P catalyst exhibits an electron transfer number (n) of 3.91, close to an ideal four‐electron pathway, and a significantly low H_2_O_2_ yield of 6.6% compared to other nanohybrids (13.4–29.2%) and all the CNHs (17.2–40.1%). It confirms efficient O─O bond cleavage and superior selectivity toward complete oxygen reduction and suppresses peroxide formation in the presence of transition metal oxides, synergistically coupled with heteroatom‐doped carbon support [6, 14, 55].

Similarly, as for ORR, for the oxygen evolution reaction (OER) (Figure 8f,g and Table S8), NiFe_2_O_4_@CNH‐N‐P exhibits the best performance under all nanohybrids, requiring a low overpotential of 290 mV at 10 mA/cm^2^, which is significantly lower than other nanohybrids and approaching RuO_2_ (260 mV). Additionally, a smaller Tafel slope (79.9 mV/dec) again indicates improved reaction kinetics. The enhanced OER is attributed to an optimized electronic structure and abundant active sites arising from the coupling between NiFe_2_O_4_ and a heteroatom‐doped carbon matrix [56]. The superior bifunctional performance of NiFe_2_O_4_@CNH‐N‐P arises from the synergistic combination of heteroatom doping, hierarchical porosity, and strong support interactions due to an intimate tethering of the ferrite particles onto the surface of the N, P doped CNH. The potential gap (ΔE) calculated after deducting the potential of the OER at 10 mA/cm^2^ (E_J_ = 10), and the half‐wave potential (E_1/2_) for the ORR is an important strategy in determining the total bifunctional activity of electrocatalysts (Figure 8h). A minimal ΔE indicates better bifunctional activity and is attractive for practical applications in rechargeable metal‐air batteries. Remarkably, the ΔE value for NiFe_2_O_4_@CNH‐N‐P is 0.73 V, which is comparable to that of commercial 20% Pt/C+RuO_2_ (0.71 V). The NiFe_2_O_4_@CNH‐N‐P catalyst exhibits a high E_1/2_ (0.79 V), low Tafel slope (56.5 mV/ dec), near four‐electron ORR pathway (n = 3.91), low peroxide yield (6.6%), and excellent OER activity (η_10_ = 290 mV).

These characteristics make it a promising candidate for rechargeable, secondary zinc‐air battery applications. The stability and rate efficiency of the catalyst NiFe_2_O_4_@CNH‐N‐P were thus analysed using multi‐step chronopotentiometry, in which the current density was sequentially increased and decreased from 10 to 100 mA cm^−2^ (Figure 8i). The catalyst displayed a stable current‐potential relationship, indicating consistent long‐term stability.

The electrochemically active surface area (ECSA) is related to the electrochemical double‐layer capacitance (C_dl_). Thus, C_dl_ was measured from the cyclic voltammetry curves attained at different scan rates in the definite potential limit (Figure S12a–f). NiFe_2_O_4_@CNH‐N‐P displayed the maximum C_dl_ and corresponding electrochemical surface area (ECSA) (Figure S12g), which is higher than for the other nanohybrids and the standard 20% Pt/C + RuO_2_. High ECSA indicates greater accessibility of more active sites for electrochemical reactions, thereby increasing electrocatalytic activity. Electrochemical impedance spectroscopy (EIS) was employed to investigate the charge transfer characteristics of the prepared catalysts. The Nyquist plots (Figure S12h) exhibit a typical semicircle in the high‐frequency region followed by a sloping line in the low‐frequency region, corresponding to charge‐transfer resistance (R_ct_) and diffusion processes, respectively. R_ct_ values were estimated from the semicircle diameters of Nyquist plots. Among all samples, NiFe_2_O_4_@CNH‐N‐P exhibits the smallest semicircle diameter, indicating the lowest charge‐transfer resistance (∼7 Ω), which is comparable to that of the benchmark 20% Pt/C + RuO_2_ catalyst. This suggests significantly enhanced interfacial charge‐transfer kinetics. In contrast, NiFe_2_O_4_@CNH and CNH‐N‐P exhibit larger semicircles, corresponding to higher R_ct_ values (∼12–15 Ω), indicating slower electron‐transfer processes.

To evaluate intrinsic kinetics, the electrocatalytic performance was normalized against the active catalyst mass loading (0.5 mg/cm^2^ for all fabricated electrodes [57]. As shown in Figure S13 and Table S6, mass‐normalized ORR polarization curves demonstrate that co‐doping the carbon support with nitrogen and phosphorus (CNH‐N‐P) significantly enhances activity over pristine and single‐doped counterparts (Figure S13a). Furthermore, integrating the spinel oxide onto the dual‐doped support NiFe_2_O_4_@CNH‐N‐P yields efficient ORR mass activity, approaching that of commercial 20% Pt/C (Figure S13b,d). In the OER (Figure S13c, Table S8), NiFe_2_O_4_@CNH‐N‐P demonstrates superior mass activity compared to single‐doped hybrids and pristine oxides, even surpassing the benchmark RuO_2_ catalyst at higher potential ranges [58]. These normalized metrics confirm that the outstanding bifunctional performance originates from intrinsically optimized charge‐transfer kinetics and synergistic interface engineering. Specifically, Figure S13d highlights the direct scaling between geometric current density (J) and mass activity at 0.8 V vs. RHE, where NiFe_2_O_4_@CNH‐N‐P achieves a remarkable ORR mass activity of −2.55 A/g. This delivers nearly five times higher intrinsic activity than its single‐doped counterparts NiFe_2_O_4_@N‐CNH and NiFe_2_O_4_@P‐CNH, closely tracking the benchmark performance of commercial 20% Pt/C (−3.35 A/g).

**SCHEME 2:**
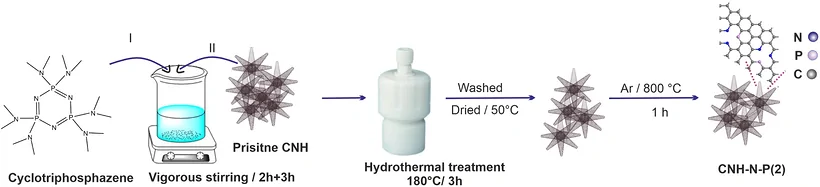
Schematics of the single‐step functionalization of CNH using molecular Hexa(N, N‐dimethylamine)cyclotriphosphazene as a single‐source precursor. For details, see Experimental Section (Supporting Information).

To validate the importance of the two‐step process for CNH functionalization, the molecular hexa(N, N‐dimethylamine)cyclotriphosphazene [59] compound was employed as a single source precursor for simultaneous N‐ and P incorporation into CNH, yielding CNH‐N‐P(2) (Scheme 2). The CNH‐N‐P(2) was characterized by XPS and ssNMR) to probe surface composition, complemented by BET surface area analysis and electrochemical evaluation toward ORR activity. The XPS survey spectrum confirms successful heteroatom incorporation (Figure S14a). High‐resolution C 1s spectra consist of sp^2^ carbon at 284.4 eV, with a minor contribution attributed to *π*–*π* ^*^ shake‐up interactions (Figure S14b). The N 1s spectrum shows peaks at 398.6, 400.6, and 402.9 eV, assigned to pyridinic N, pyrrolic N, and oxidized nitrogen species, respectively (Figure S14c). The P 2p peaks exhibit a characteristic doublet at 132.8 and 133.9 eV, attributed to P‐C or P‐N in close resemblance to the results obtained in the two‐step synthesis procedure (Figure S14d) [38]. The ssNMR spectrum, exhibits a broad resonance centered around 2.2 ppm, characteristic of phosphorus substitution in a carbon environment. The broad line shape reflects the structural disorder and distribution of heteroatom N and P doping (Figure 7d) [38]. The ^31^P NMR spectrum of CNH‐N‐P(2) is relatively noisier than those of other samples due to its lower phosphorus content and fewer scans.

Despite the successful introduction of N and P heteroatoms, the single‐precursor approach yields lower N and P surface concentration than the sequential functionalized CNH‐N‐P material (Table S4). Evidently, the two‐step process involving successive thermal treatments promotes the formation of a higher density of active sites. Notably, the initial N‐functionalization step facilitates subsequent P incorporation as also evidenced by increased N and P atomic percentages obtained from XPS analysis. In addition, the CNH‐N‐P(2) exhibits lower adsorption capacity, less pore volume, and low surface area characteristics as compared to CNH‐N‐P (Table S3 and Figure S14e), suggesting reduced active surface accessibility, probably resulting in slower exchange and mass transfer kinetics. This is indeed substantiated by a better ORR activity of NiFe_2_O_4_@CNH‐N‐P as compared with NiFe_2_O_4_@CNH‐N‐P(2) (Figure S14f), and in accord with a higher surface area (Table S3) and higher atomic percent of N and P in CNH‐N‐P (Table S4).

The improved performance of NiFe_2_O_4_@CNH‐N‐P is credited to the synergistic effect of heteroatom doping (N and P) and the presence of NiFe_2_O_4_, which enhances electrical conductivity and facilitates rapid charge transport across the electrode‐electrolyte interface. The mixed‐valence Ni^2+^ / Ni^3+^ and Fe^3+^ states are effective active sites for the adsorption and desorption of reaction intermediates. Charge transfer between such cations with different valence states in octahedral sites is greatly enhanced [5]. On the other side, N and P doping into the carbon lattice generates defects and disrupts charge neutrality, generating positively charged carbon atoms adjacent to N/P dopants that lower the adsorption energy of oxygen intermediates during the ORR [16, 17, 26]. The direct chemical coupling between NiFe_2_O_4_ and CNH‐N‐P results in interfacial collaboration, as confirmed by XPS peak shifts and HR‐TEM. This interfacial coupling facilitates rapid interfacial charge transfer, modulates the Ni/Fe active sites, and significantly lowers the activation energy barriers for both ORR and OER compared to either component alone. To further support our claim, we compared the ORR performance of pure NiFe_2_O_4_, CNH‐N‐P, a physical mixture of NiFe_2_O_4_ and CNH‐N‐P (44 wt.% NiFe_2_O_4_ + 56 wt.% CNH‐N‐P, matched to the TGA‐determined composition of NiFe_2_O_4_@CNH‐N‐P), and the synthesized NiFe_2_O_4_@CNH‐N‐P nanohybrid (Figure S15a). Pure NiFe_2_O_4_ shows the weakest activity, and pure CNH‐N‐P performs considerably better in terms of onset potential, half‐wave potential, and limiting current density (Table S6). The physical mixture (of the two components) shows a limiting current density comparable to that of pure CNH‐N‐P; however, it lags in onset and half‐wave potentials, indicating that simply combining the two materials without chemical coupling does not meaningfully enhance performance beyond that of the better‐performing individual component. In contrast, the NiFe_2_O_4_@CNH‐N‐P composite substantially outperforms all three control samples, exhibiting the most positive onset and half‐wave potentials and the highest limiting current density.

### 2.3 NiFe_2_O_4_@CNH, NiFe_2_O_4_@N‐CNH, NiFe_2_O_4_@P‐CNH, and NiFe_2_O_4_@CNH‐N‐P as Bifunctional Cathode Materials in Secondary Zinc‐Air Batteries

**FIGURE 9:**
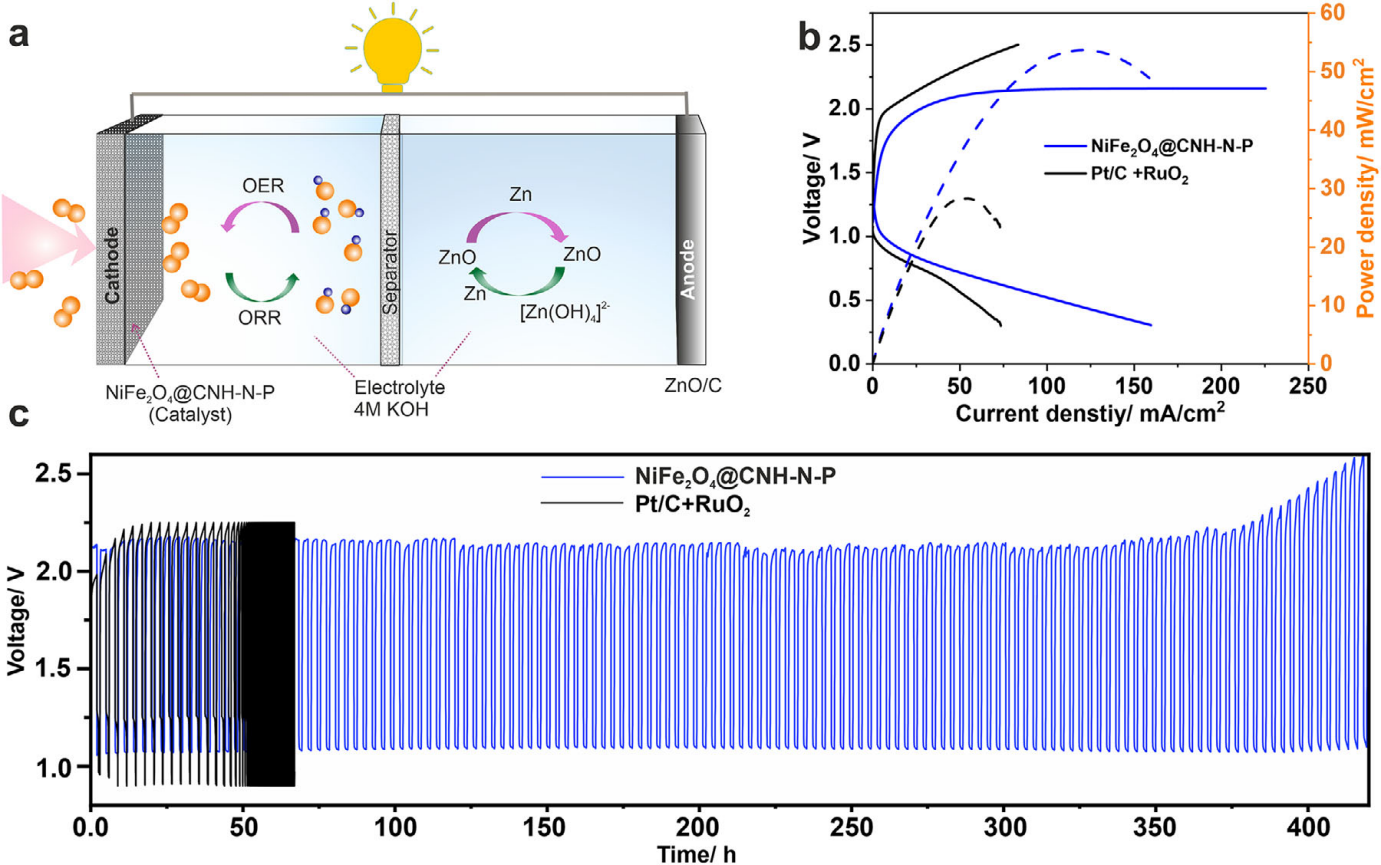
(a) Schematic representation of the general set up of a rechargeable Zinc‐air battery, (b) charge–discharge polarization curves of the zinc‐air batteries using NiFe_2_O_4_@CNH‐N‐P and 20% Pt/C−RuO_2_ as air electrodes, respectively, along with corresponding discharge power density of the catalyst, (c) charge–discharge curves of NiFe_2_O_4_@CNH‐N‐P and 20% Pt/C + RuO_2_ at the current density of 0.55 mA/cm^2^/3 h per cycle.

To establish the practical applications of ferrite/CNH nanohybrids as bifunctional electrocatalysts in rechargeable ZABs, we studied an in‐house Swagelok‐based secondary ZAB. Therein, the nanohybrids are each embedded in a diffusion layer, with an air‐open cathode and a ZnO/C hybrid as the anode, together with 4.0 m KOH as the electrolyte. (see Figure 9a and Experimental Section (Supporting Information) for details) [14, 60]. The ZnO/C anode comprises ZnO nanoparticles embedded in a structured porous carbon framework derived from the thermal pyrolysis of MOF‐5. We recently reported a MOF‐5‐derived ZnO/C anode and its use in rechargeable zinc‐air batteries [9, 17]. This ZnO/C anode outperforms conventionally used zinc foil and helps to determine the real specific discharge capacity, which is not possible when using a bare zinc anode. The ZnO/C anode provides efficient zinc availability due to its 3D porous structure, thereby improving zinc utilization and allowing the determination of the specific discharge capacity. It enables regenerative ZnO conversion by addressing issues of dendrite formation, ZnO dissolution, and electrode passivation. A ZAB using a 20% Pt/C + RuO_2_ (1:1 mass ratio) as a bifunctional standard catalyst in the air electrode serves as a reference.

The open‐circuit voltage of ZAB built with NiFe_2_O_4_@CNH‐N‐P is 1.40 V, which is higher than that of ZAB with 20% Pt/C + RuO_2_ (1.21 V). Given the good oxygen redox activity, the polarization curves of ZAB recorded with NiFe_2_O_4_@CNH‐N‐P as the cathode catalyst exhibit a smaller charge–discharge voltage gap than those of 20% Pt/C + RuO_2_ (Figure 9b). The maximum power density derived from polarization curves for the ZAB built with NiFe_2_O_4_@CNH‐N‐P is 54 mW/cm^2^, nearly twice the 28 mW/cm^2^ of the ZAB with 20% Pt/C+RuO_2_ as the bifunctional catalyst. To assess the long‐term durability, the galvanostatic charge–discharge profiles at a current density of 0.55 mA/cm^2^ for the ZAB with NiFe_2_O_4_@CNH‐N‐P and 20% Pt/C+RuO_2_ are compared (Figure 9c). The voltage gap during constant‐current discharge measurements is smaller for battery devices with NiFe_2_O_4_@CNH‐N‐P as cathode, confirming its better rate durability. After continuous charging and discharging for over 300 h, the voltage gap remained constant. In comparison, 20% Pt/C + RuO_2_ displayed rapid performance degradation after 50 h. The zinc‐air battery based on NiFe_2_O_4_@CNH‐N‐P as the cathode exhibits better cycling performance, including depth‐of‐discharge (DOD) and discharge capacity retention. The maximum discharge capacity of NiFe_2_O_4_@N‐CNH‐N‐P is 293.5 mAh/g (DOD 35.70%) and shows constant behavior over ∼100 cycles. The cycling stability measurements further highlight the robustness of the N, P‐doped nanohybrid catalyst. NiFe_2_O_4_@CNH‐N‐P maintains stable charge–discharge cycling for ∼more than 100 cycles, whereas NiFe_2_O_4_@CNH, NiFe_2_O_4_@N‐CNH, and NiFe_2_O_4_@P‐CNH show significantly shorter lifetimes of ∼31, ∼46, and 53 cycles, respectively, with non‐uniform capacity variation (Table S9, Figure S16). To evaluate the chemical and mechanical stability of the catalyst, inductively coupled plasma optical emission spectrometry (ICP‐OES) was performed on the electrolyte after long‐term charge–discharge cycle. As shown in Table S10, no detectable nickel ions were found in the post‐cycled electrolyte. Furthermore, the iron content (0.259 mg/L) remained identical to the baseline value of the fresh, unreacted 4 mol/L KOH solution. These results definitively demonstrate that negligible metal leaching occurs during operation, confirming the robust structural integrity of the catalyst.

To assess the structural, morphological, and compositional stability of the catalyst after extended operation, we performed post‐cycling characterizations using high‐resolution XPS, EDX quantification, and HRTEM imaging. (Figure S14). The XPS analysis of the electrode, both in the pristine state and after cycling, was performed to assess the catalyst's structural stability (Figure S15b,c). The Ni 2p and Fe 2p spectra show no significant new peaks or shifts after cycling, indicating that the composite retains its structural and chemical integrity over extended operation. Quantitative analysis of the Ni:Fe ratio shows only a modest change, from 1:2.4 in the pristine electrode to 1:2 after cycling, consistent with good compositional stability. The overall decrease in peak intensity after cycling is attributed to a residual KOH layer on the electrode surface, which was only partially removed despite scratching selected areas prior to measurement. We also note that the Fe 2p_3/2_ satellite feature overlaps with the Sn 3p_3/2_ signal, originating from the tin current collector used on the anode side, and this overlap should be considered when interpreting the Fe 2p region. The N 1s and P 2p signals were notably weak and noisy in these measurements, likely due to the low nitrogen and phosphorus content of the electrode surface after cycling and washing. Comparative energy‐dispersive x‐ray spectroscopy (EDX) analysis between the fresh electrode (Figure S15d) and post‐cycled electrode (Figure S15e) confirms compositional durability. On the pristine catalyst, elemental mapping/quantification yields Ni (2.33 at.%) and Fe (5.51 at.%), reflecting a pristine Ni: Fe ratio close to 1:2.36. Post‐cycling EDX shows Ni (0.59 at.%) and Fe (1.27 at.%), yielding a Ni: Fe ratio of 1:2.15, demonstrating that the stoichiometry of the active metal centers is well preserved. Signals corresponding to electrolyte‐derived K (18.26 at.%) and Zn (15.63 at.%) are naturally observed on the cycled surface due to residual KOH and zincate species from the ZAB reaction environment. Post‐cycling high‐resolution transmission electron microscopy (HRTEM, Figure S15f) demonstrates that the NiFe_2_O_4_@CNH‐N‐P catalyst retains its distinct nanocube morphology anchored on the carbon nanohorn matrix without severe agglomeration or collapse. The inset HRTEM image clearly reveals well‐preserved lattice fringes with interplanar d‐spacings of 0.48 and 0.25 nm, which correspond directly to the (111) and (311) crystal planes of the spinel NiFe_2_O_4_ phase, confirming that the crystalline spinel core remains intact throughout testing. Taken together, these combined XPS, EDX, and HRTEM results confirm that the NiFe_2_O_4_@CNH‐N‐P composite retains its structural and chemical composition after cycling, with no evidence of significant degradation.

Compared with previously published zinc‐air batteries employing ZnO/C as the anode, the present catalyst demonstrates superior electrochemical performance, delivering a specific discharge capacity of 293.5 mAh/g and stable operation over 100 charge–discharge cycles (300 h+). Previous reports with CoFe_2_O_4_@N‐CNH [14] as cathode catalysts with ZnO/C anode reported a maximum discharge capacity of 280 mAh/g, with a cycle life of ∼55.

**FIGURE 10:**
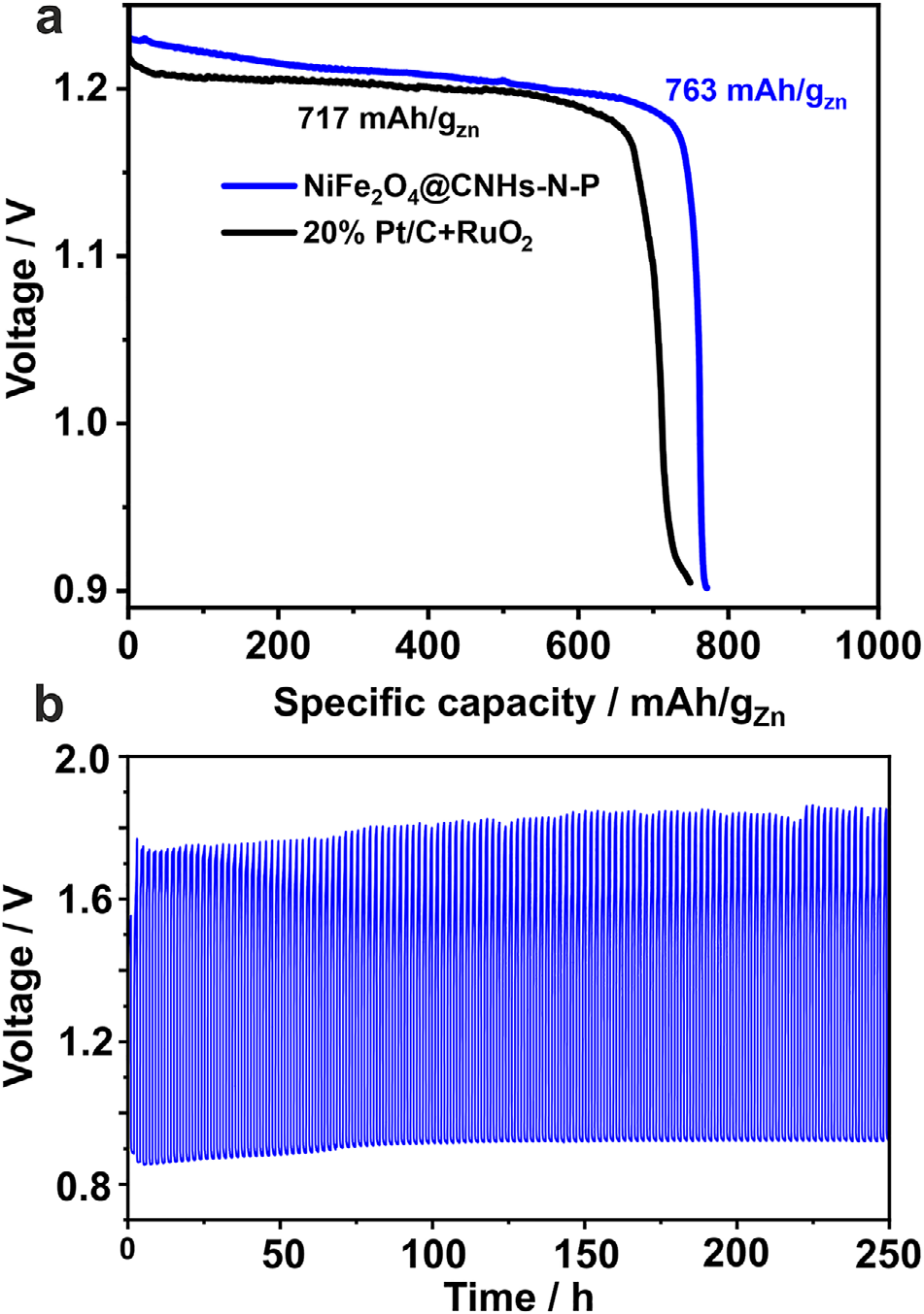
Zinc–Air Battery (ZAB) performance with zinc foil as anode. (a) Galvanostatic discharge curves of ZAB assembled with NiFe_2_O_4_@CNH‐N‐P and benchmark 20% Pt/C + RuO_2_ as the air cathode catalysts, at a discharge current density of 5 mA/cm^2^, using zinc foil (13 mm diameter, 0.16 mm thickness) as the anode, (b) long‐term galvanostatic charge–discharge cycling of a rechargeable ZAB assembled with NiFe_2_O_4_@CNHs‐N‐P as the bifunctional air cathode and zinc foil (13 mm diameter, 0.16 mm thickness) as the anode, cycled at a current density of 5 mA/cm^2^ (with each charge–discharge cycle lasting 1 h), over a total testing period of 250 h.

To put our findings in perspective, we compared them with a ZAB using zinc foil anodes. Zinc foil was utilized as a benchmark anode to establish a comparative baseline against established literature, specifically to evaluate the intrinsic activity of the NiFe_2_O_4_@CNH‐N‐P cathode. The galvanostatic constant current (5 mA/cm^2^) discharge profiles with NiFe_2_O_4_@CNH‐N‐P as cathode and zinc foil as anode display a high specific capacity of 763 mAh/g_Zn_, significantly exceeding that of 20% Pt/C‐RuO_2_ as cathode catalyst (717 mAh/g_Zn_), both determined relative to consumed zinc metal (Figure 10a). The extended discharge plateau suggests stable oxygen reduction kinetics and efficient utilization of active sites. This improvement is attributed to a hierarchical, stable porous structure that ensures efficient oxygen diffusion and electrolyte penetration, as well as an optimized electronic structure induced by N, P co‐doping. The galvanostatic cyclization performance of ZAB with the standard zinc‐foil anode exhibits durability, with a cyclic life of over 250 h (Figure 10b). Thus, compared to standard zinc foil, which suffers from severe passivation and irreversible dendrite growth that limits reversibility, the ZnO/C composite anode offers a significant advantage. Its unique structure accommodates volume expansion during cycling and promotes uniform zinc deposition, thereby inhibiting dendrite formation and enhancing overall battery lifespan. Furthermore, a few previously published reports describe NiFe_2_O_4_‐carbon composites as bifunctional catalysts for zinc‐air batteries [6, 7, 8, 10, 12, 13]. A comparison of the zinc‐air battery performances reported for various NiFe_2_O_4_‐carbon composite catalysts is provided in Table S11. As evident from the comparison, our synthesized NiFe_2_O_4_@CNH‐N‐P catalyst delivers a favorable combination of properties that, taken together, are better than those of existing state‐of‐the‐art NiFe_2_O_4_‐based carbon composites reported in the literature. A bifunctional gap (ΔE) of 0.73 V, competitive with or better than most listed catalysts. The OER overpotential (η_10_) of 290 mV is among the lowest in the comparison set, indicating balanced bifunctional (ORR+OER) activity rather than optimization for a single reaction. Most notably, superior long‐term ZAB cycling stability of 300+ h, exceeding every other entry in the table, several of which report stability only in the 80–200 h range.

## 3 Conclusion

A series of NiFe_2_O_4_‐decorated carbon nanohorn (CNH) hybrids with controlled tetrel‐heteroatom doping (N‐ and P‐single doping and N, P co‐doping) was successfully synthesized and systematically investigated. Structural characterization by XRD and HRTEM confirms the formation of crystalline spinel NiFe_2_O_4_ nanoparticles uniformly anchored on the CNH framework, while TEM analysis reveals intimate interfacial contact between the ferrite particles and the CNH surface, enabling their high dispersion on the surface. TGA results indicate improved thermal stability of the hybrid structures, and XPS analysis demonstrates the successful incorporation of N and P dopants, the formation of NiFe_2_O_4,_ and electronic interactions between NiFe_2_O_4_ and the carbon framework. Electrochemical studies reveal that NiFe_2_O_4_@CNH‐N‐P exhibits outstanding bifunctional catalytic activity, with a high ORR‐half wave potential (0.79 V), low Tafel slope (56.5 mV/dec), a near four‐electron transfer pathway (n = 3.91), and minimal peroxide yield (6.6%). In addition, it exhibits superior OER performance with a low overpotential (290 mV at 10 mA/cm^2^) and favorable kinetics. The combined enhancements are attributed to synergistic effects of heteroatom incorporation‐induced e.g., hierarchical porosity, and strong metal ferrite/CNH‐support interactions, which collectively optimize adsorption energy and charge transfer. When implemented in a rechargeable zinc‐air battery, NiFe_2_O_4_@CNH‐N‐P delivers a high specific capacity (293.5 mAh/g) in connection with a ZnO/C anode, excellent power density, and remarkable cycling stability (over 100 cycles), outperforming the benchmark 20% Pt/C‐RuO_2_ bifunctional catalyst (35 cycles). The improved durability compared to other NiFe_2_O_4_@CNH‐N‐P hybrids highlights the structural robustness and catalytic stability of the co‐doped system.

Overall, this work demonstrates that the rational design of heteroatom‐doped carbon‐metal oxide hybrids is an effective strategy for developing low‐cost, high‐performance bifunctional electrocatalysts, with significant potential for next‐generation energy conversion and storage technologies.

## Author Contributions

**Sudheer Kumar Yadav**: investigation, writing – original draft, writing – review and editing, formal analysis, data curation, methodology, validation, visualization. **Vanessa Trouillet**: investigation, writing – review and editing, formal analysis. **Jonas Lins**: investigation, validation, writing – review and editing. **Torsten Gutmann**: investigation, writing – review and editing. **Jörg J. Schneider**: conceptualization, funding acquisition, writing – review and editing, methodology, writing – original draft, project administration, supervision, resources.

## Conflicts of Interest

The authors declare no conflicts of interest.

## Supporting information




**Supporting File**: advs77749‐sup‐0001‐SuppMat.docx.

## Data Availability

The data that supports the findings of this study are available in the supplementary material of this article.
