# Supplementary material for: Nickelferrite on Pnictogen Functionalized Carbon Nanohorns. Bifunctional ORR and OER Catalysts in Rechargeable Zinc‐Air Batteries
Source: Adv Sci (Weinh). 2026 Sep 27:e77749. Online ahead of print. doi: 10.1002/advs.77749 (PMC13616351; doi:10.1002/advs.77749)
Supplement: Supplementary file 1 [file ADVS-9999-e77749-s001.docx]

Supporting Information

**Nickelferrite on pnictogen functionalized carbon nanohorns. Bifunctional ORR and OER catalysts in rechargeable zinc-air batteries.**

Sudheer Kumar Yadav, ^[a]^ Vanessa Trouillet, ^[b]^ Jonas Lins, ^[c]^ Torsten Gutmann, ^[c,#]^ Jörg J. Schneider*^[a]^

*^[a]^Fachbereich Chemie, Eduard-Zintl-Institut für Anorganische und Physikalische Chemie,*

*Technische Universität Darmstadt*

*Peter-Grünberg-Strasse 12, 64287 Darmstadt, Germany*

*^[b]^Institute for Applied Materials (IAM-ESS) and Karlsruhe Nano Micro Facility (KNMFi)*

*Karlsruhe Institute of Technology (KIT)*

*Hermann-von-Helmholtz-Platz 1, B 321, 76344 Eggenstein-Leopoldshafen, Germany*

*^[c]^ Eduard-Zintl-Institute for Inorganic and Physical Chemistry, Technical University Darmstadt,*

*Peter-Grünberg-Straße 8, 64287 Darmstadt, Germany*

*^#^Current address: Department of Chemistry, Physical Chemistry, Paderborn University, Warburger Straße 100, 33098, Paderborn, Germany*

**EXPERIMENTAL PROCEDURES**

**Functionalization of CNHs**

**N-CNH**

A simple thermal treatment of CNHs and urea at higher temperature was used for the synthesis of the N-CNHs. 50 mg of CNHs was mixed with 250 mg of urea using a mortar and pestle followed by annealing at temperatures 800 °C for 1 h in argon atmosphere. The resulting nitrogen-doped CNH was then designated as N-CNH.

**P-CNH**

100 mg of CNHs was dispersed into 80 mL water. After the solution was well dispersed with stirring for 2h, 500 g of phytic acid (PA, 50 % (w/w) in H_2_O) (also known as inositol hexaphosphate) was added into the above solution and for vigorous stirring for 3 hours. Next step, the solution was transferred to 100 mL Teflon lined stainless-steel autoclave and hydrothermally treated at 180 °C for 3 hours. Afterwards, it was cooled down to room temperature and solution mixture was centrifuged, washed and dried to get PA@CNH. The P-CNH was prepared by annealing PA@CNH in the tube furnace at 800 °C for 1 h under an argon atmosphere. The heating rate and gas flow rate were set to be 10 °C/ min and 50 mL /min, respectively.

**CNH-N-P**

Phosphorous functionalization of N-CNH. Preparation method was followed as for P-CNH using N-CNH as the starting material.

**Treament of CNH with Hexa(N,N-dimethylamine)cyclotriphosphazene, P_3_N_3_(C_2_H_6_N)_6_)**

**(One step N, P-functionalization CNH)**

The cyclotriphosphazene was synthesized as reported.^[1]^ The product obtained was sublimed to obtain further purified cyclotriphosphazene. The single step functionalization process of CNH includes following steps: 250 mg of cyclic phosphazene compound was dissolved in 80 mL of water by vigorous stirring for 2 hours. 100 mg of CNH was added to the above solution and further stirred for 3 hours. Finally, the solution was transferred to 100 mL Teflon lined autoclave stainless-steel and treated at 180 °C for 3 h. Afterwards, it was cooled down to room temperature and solution mixture was centrifuged, washed and dried at 50 °C for overnight. Further, the obtained powder was annealed in the tube furnace at 800 °C for 1 h under an argon atmosphere to obtain N, P-doped CNH (CNH-N-P(2)). The heating rate and gas flow rate were set to be 10 °C/ min and 50 mL /min, respectively.

**Preparation of Fe(NH_2_CONH_2_)_6_(NO_3_)_3_ and Ni(NH_2_CONH_2_)_6_(NO_3_)_2_**

*Synthesis of Fe(NH_2_CONH_2_)_6_(NO_3_)_3_:* Hexakis(urea)iron(III)nitrate was prepared following a documented method:^[2]^ Initially, 4.85 grams (equivalent to 12 mmol) of iron nitrate nonahydrate was dissolved in 250 mL of ethanol, while 6.006 grams (equivalent to 100 mmol) of urea was dissolved in 100 milliliters of ethanol. Subsequently, these two solutions were combined and stirred for a duration of 2 hours. During this time, a yellow precipitate formed, which was subsequently separated via filtration. The resulting precipitate was then washed with ethanol and subjected to vacuum drying, resulting in a final yield of 5.29 grams (equivalent to 73%)*.*

*Synthesis of Ni(NH_2_CONH_2_)_6_(NO_3_)_2_:* Hexakis(urea)nickel(II) nitrate was prepared through a co-precipitation method.^[3]^ Initially, 3.9 g (65 mmol) of urea was dissolved in 100 mL of n-butanol, and 2.91 g (10 mmol) of nickel(II) nitrate hexahydrate was added. After stirring for two hours, a green precipitate was obtained. The precipitate was filtered, washed with n-butanol, and dried under vacuum (3.58 g,6.6 mmol, 66% of theory).

*Synthesis of NiFe_2_O_4_@CNHs:* A single-step hydrothermal method was used to prepare the nickel ferrite@CNHs nanohybrid. Stoichiometric amounts of single-source molecular precursors (nickel urea complex (1 mmol) and iron urea complex (2 mmol), as well as 50 mg of CNH (CNH, N-CNH, P-CNH, CNH-N-P) were well disseminated in 80 mL of water for 2 hours by continuous stirring. Stirring aids in the dissolution of urea complexes and the uniform distribution of CNH particles throughout the solvent. The mixture was transferred to 100 mL Teflon-lined stainless-steel autoclave and kept in oven maintained at 180 °C for 2 hours. After two hours autoclave was cooled to room temperature. The product obtained was washed with water and overnight dried in oven at 50 °C.

**Characterization**

X-ray diffraction was performed on Rigaku Mini-Flex 600, D/teX Ultra Detector with CuKα radiation (λ=0.1540598 nm). Pattern were recorded over 2θ range of 10°-90° at a scan rate of 0.5°/min. Thermogravimetric analysis was conducted on a Netzsch TG 209 F1 to determine carbon percentage at heating rate of 10 °C/min in oxygen atmosphere (O_2_ flow rate of 25 mL/min). Raman spectroscopy was performed using high-resolution Raman spectrometer (Thermo Scientific, model DXR3), with a diode-pumped solid state (DPSS) laser 514 nm laser as an excitation source. The structure and surface characteristics were obtained using Scanning electron microscope (Philips XL30 FEG) and a FEI Tecnai G2 F20 Transmission electron microscope. The surface area characteristics were determined by using a Quanta chrome 3000e at 77 K. Before each measurements samples were degassed in a vacuum for 24 hours at 150 °C. The results were calculated using the NOVA (version 10) software. A K-Alpha+ XPS spectrometer (ThermoFisher Scientific) was used for the XPS measurements. The Thermo Avantage software was used for data acquisition and processing. All samples were analyzed with a 400 μm spot size micro focused, monochromatic Al K X-ray source. During the analysis, the K-Alpha+ charge compensation system was used, which used electrons with an energy of 8 eV and low-energy argon ions to avoid any localized charge build-up. The spectra were quantified using Scofield sensitivity factors^[4]^ and one or more Voigt profiles (BE uncertainty: ±0.2 eV). All the spectra were referenced to C 1s peak (C-C, C-H) at 285.0 eV binding energy regulated by the photoelectron peaks of metallic Cu, Ag, and Au, respectively.

^31^P NMR measurements were performed on a Bruker Avance III HD 300 MHz spectrometer at 7 T corresponding to frequencies of 300.11 MHz for 1H and 121.49MHz for ^31^P. The spectrometer was equipped with a 4 mm broadband 1H/X probe. Samples were packed in a 4 mm zirconium rotor. One pulse experiment for ^31^P were performed using a 90° excitation pulse of 6 µs and 4 s recycle delay. The spinning rate for MAS varied between 5 kHz and 6.25 kHz. All solid samples were measured without proton decoupling. Liquid samples (H_3_PO_4_ reference, phytic acid) were measured without MAS using the hpdec pulse sequence and 8 scans.

Spectra were referenced to H_3_PO_4_ (0 ppm) as external standard and were analyzed using the MestReNova software package.

**Electrochemical measurements**

*ORR, EIS and OER activity measurements:* Electrochemical studies were performed employing a Biologic potentiostat (Model VMP3) and electrochemical workstation by executing numerous practices such as cyclic voltammetry (CV), linear sweep voltammetry (LSV) and chronopotentiometry (CP). RDE and RRDE analysis were performed over a BluRev RC-10K Rotating Disk and Ring-Disk Electrode. A three-electrode setup was assembled for the analysis: Catalyst staged on glassy carbon disk (GC) as working electrode, Pt rod as the counter electrode and Hg/HgO as the reference electrode. All potentials are reported versus RHE, calculated from Hg/HgO using equation E_RHE_ = E_Hg/HgO_ + 0.098 + 0.059 × pH (pH = 13 for 0.1 M KOH).

For the catalyst slurry preparation, 3.50 mg of catalyst, 140 µl of ethanol, 10 µl of Nafion (5%) and 100 µl of deionized water were dispersed for 30 min (ultra-sonicated). A 2.5 µl slurry was drop mounted on the surface of GC disk of RDE (or RRDE) and dried at room temperature to be used as working electrode with catalyst loading of 0.50 mg/cm^2^. For comparison, commercial catalysts 20 wt.% Pt/C (or 20 wt.% Pt/C: RuO_2_ = 1:1, mass ratio) were used with same catalyst loading.

The ORR analysis was performed in 0.1 M KOH, sweeping from 0.3 V to -0.7 V vs. Hg/HgO reference electrode at a scan rate of 10 mV/ s with different electrode rotation at 400, 600, 900, 1600, and 2500 rpm, and O_2_ gas was purged continuously into the solution at a flow rate of 25 sccm through an 8 mm fritted tube (Lenz Laborglas GmbH & Co).^[5,6]^ The Tafel slope was derived from the equation η= *b* log *j* + *a*, where η, *b*, and *j* are the overpotential, Tafel slope, and current density, respectively. CV measurements were conducted by varying the scan rates from 10 to 35 mV/s to calculate the electrochemical double-layer capacitance (C_dl_). The electrochemical double-layer capacitance (C_dl_) is equal to the ratio of double-layer charging current and the product of the scan rate (v):

𝐶_𝑑𝑙_ = i/v

Thus, a plot of i as a function of v yields a straight line with a slope equal to C_dl_.

Further, the electrochemical active surface area (ECSA) of the catalyst was calculated as:

𝐸𝐶𝑆𝐴 = 𝐶_𝑑𝑙_/𝐶_𝑠_

Where 𝐶_𝑑𝑙_ is the specific capacitance of the electrode double layer (mF); v (V/s) is the scan rate; 𝐶_s_ is the specific capacitance. According to the literatures, the C_s_ of 0.1 M KOH at 25 °C is 0.06 mF/cm^2^.^[7]^

Electrochemical impedance spectroscopy (EIS) was measured 200 mV with a frequency range of 200 KHz−0.1 Hz.

For the RRDE tests, the disk electrode was scanned at a rate of 10 mV/s, and the ring potential was held at 0.3 V (vs. Hg/HgO). The following equations were used to calculate the percentage of peroxide species in relation to total oxygen reduction products and the number of electrons involved during the ORR process:

$${HO}_{2}^{-}=200\frac{I_{r}/N}{I_{d}+I_{r}/N}$$

$$n=4\frac{Id}{I_{d}+I_{r}/N}$$

Here, I_d_ and I_r_ are the disk and ring current, respectively, and N is the current collection efficiency of the Pt ring (N= 0.46) from the reduction of K_3_Fe(CN)_6_.^[8]^ Only the rotating-disk electrode (RDE) was used for the OER test, and the working electrode was scanned from 0.3 to 1.2 V (vs. Hg/HgO) at a scan rate of 10 mV/s in an O_2_-saturated electrolyte 0.1 M KOH, while spinning at 1600 rpm. To rule out geometric loading effects and evaluate intrinsic kinetics, the electrocatalytic performance was normalized against the active catalyst mass loading (0.5 mg/cm^2^) for all fabricated electrodes following method mass activity (A/g) = Geometric current density (mA/cm^2^)/ catalyst mass loading (0.5 mg/cm^2^).^[9,10]^

*Zinc-air battery studies*

*Preparation of Air electrode/Gas diffusion electrode*: The catalyst slurry was prepared by dispersing 5 mg of catalyst in a solution of 56 mg of 1 wt. % Polyvinylidene fluoride (PVDF) in N-Methyl-2-pyrrolidone (NMP). The slurry makeup (ratio by weight of 90% catalyst and 10% PVDF) was kept same for all the analysis. The slurry was film layered on the gas diffusion layer (19 mm, TEFLON®-coated carbon paper, Sigracet® GDL 39 BB, SGL) and dried overnight at 50°C. The catalyst-spread area was 2 cm^2^ (16 mm diameter) of the electrode, resulting in a catalyst loading of 2.5 mg/cm^2^.*Preparation of composite anode*: The details of ZnO/C composite anode fabrication is described elsewhere.^[11]^ In brief, hierarchically porous ZnO/C composite was synthesized by controlled pyrolysis of MOF-5. MOF-5 was put in a quartz boat positioned on an in house made magnetic transfer arm and placed into the hot zone of a tube furnace operating at 550 °C with a constant argon flow of 50 sccm. After 10 minutes, the sample was cooled to room temperature using a continuous argon flow. For composite anode fabrication ZnO/C composite is dispersed in a 5 wt.% solution of fumion®-FAA3 (Fumatech) in N-Methyl-2-pyrrolidone (NMP), followed by the application of a film of the prepared dispersion onto a cylindrical tin foil (Sn, 99.9% ,13 mm diameter and 15 μm thickness). The fabricated electrodes have an average of 2.5 mg of ZnO, and fumion®-FAA3 is used as a binder in the ratio ZnO/C: FAA3 = 80: 20.

*ZAB assembly and measurements:* We utilized an internally manufactured two-electrode testing apparatus for subsequent electrochemical analysis. This apparatus consisted of several components: The cell casing was constructed using a Swagelok® connector (PFA/ASTM D3307 Type I material) cylindrical in shape. For the cathode current collector, we employed a cylindrical, perforated metal plate fitted with a pin electrode. The pin electrode had a diameter of 20 mm, a length of 50 mm, and was made of stainless steel (AISI316). On the anode side, we used a fully tin stamp electrode with a diameter of 13 mm and a shaft length of 100 mm, composed of 99.9% tin. This tin stamp current collector was secured in a guide holder (fiber-glass-reinforced polyamide 6.6 material) measuring 13 mm in diameter, 55 mm in length, and featuring a 5 mm bore. To affix the gas diffusion electrode directly onto the Swagelok® connector, with the catalyst layer facing the interior of the cell casing, we employed an adhesive tape (3M VHB tape 4905). The cathode's stainless steel current collector was securely fastened with a screw cap, ensuring a constant electrical connection with the gas diffusion electrode. To separate catalyst layer from the anode side composite electrode we used two cylindrical laminated separator discs of Celgard® 5550 (surfactant coated, diameter of 14 mm and thickness of 110 μm). The composite anode maintains physical contact with the tin stamp electrode, creating a direct link to the outside of the cell. Both electrodes were housed within the inner cell case and were surrounded by an aqueous alkaline electrolyte (2 mL, 4 mol/L KOH). The test cell was set up vertically, with electrodes positioned horizontally. The charge-discharge cycling stability was tested using a VMP3 multichannel potentiostat (Bio-Logic) at ambient temperature. (other measurements details discussed in main text). Zinc-air cell builds with ZnO/C displaying initial discharge state OCV of 1.40 V were galvanostatically charged to 2.25 V and discharged to 0.9 V (GCPL, galvanostatic cycling with potential limitation) using air as oxygen supply. In the case of ZnO/C cell, the active material's specific discharge capacity (mAh/g) is determined by multiplying the discharge time (in hour) by the applied current (0.55 mA/cm^2^), considering the quantity of ZnO (2.5 mg) in the system for the relevant half-cell reaction.^[11]^ Further, the and power density (mW/cm^2^) is determined by polarization measurements.^[12]^ The DOD reported here is depth of discharge of zinc as a percentage of the theoretical capacity of the [zinc electrode](https://www.sciencedirect.com/topics/engineering/zinc-electrode) (819 mAh/g) calculated according to Parker et al.^[11,13]^ DOD_practical_ = (Specific discharge capacity/ theorectical capacity) x 100.

*Zinc foil electrode for primary (galvanostatic constant current) and secondary Zn–air cell testing:* A zinc foil disc (13 mm diameter, 0.16 mm thickness, mass ≈ 0.152 g) was used as the anode for both primary and secondary (rechargeable) Zn–air battery testing. Prior to cell assembly, the zinc foil was polished with fine sandpaper, rinsed sequentially with ethanol and deionized water to remove surface oxides and residual contaminants, and then dried under ambient conditions.

The zinc foil anode was assembled against the NiFe_2_O_4_@CNHs-N-P (or benchmark 20% Pt/C + RuO₂) air cathode in a two-electrode Zn–air cell configuration. The same aqueous alkaline electrolyte (4 mol/L KOH) and the same cell assembly (separator, cathode loading, air-access geometry, testing temperature) were used for both primary and secondary cell tests, consistent with the conditions described for the ZnO/C anode above. For primary cell testing, cells were discharged galvanostatically at  5 mA/cm² until reaching the cutoff voltage of 0.9 V, from which specific capacity (mAh/g_Zn_) were determined with respect to consumed zinc. The cells were evaluated down to a cutoff voltage of 0.9 V. This cutoff was intentionally selected because Zn–air batteries typically operate at discharge voltages of approximately 1.0–1.1 V ^[14]^ under practical galvanostatic conditions. Thus, a 0.9 V cutoff provides a sufficiently low potential limit to capture the principal discharge regime while avoiding unnecessarily deep discharge. We maintained a cutoff voltage of 0.9 V throughout the manuscript for both galvanostatic cycling with potential limitation (GCPL) and galvanostatic constant-current measurements. For secondary (rechargeable) testing, cells were cycled galvanostatically at 5 mA/cm^2^ with a fixed time of discharge (60 min)  and charge (60 min) steps. With a cell voltage variation similar to that of the ZnO/C cell.

ICP-OES analyses were performed on the KOH electrolyte after post long term charge-discharge cycle using AMETEK/Spectro ICP-OES Genesis and analysed using Spectro ICP-Analyzer Pro. Each samples were diluted to 50 times with HPLC gradient grade water (Fischer Scientific) for the measumrent.

**Figure S1.** (a) FT-IR spectra of urea and metal urea complexes, and (b) TGA of a mixture of the two complexes Ni and Fe urea complexes in stoichiometric ratio. The in-set shows the XRD pattern of remnant obtained after TGA identifying it as the pure NiFe_2_O_4_ inverse Spinel phase.

The FT-IR. In comparison to pristine urea, which exhibits a characteristic C=O stretching vibration at ~1675 cm⁻¹, both metal complexes show a pronounced shift of this band to lower wavenumbers (~1625 cm⁻¹), indicating coordination of the carbonyl oxygen to the metal centers. Additionally, the C–N stretching vibration shifts from ~1459 cm⁻¹ in free urea to ~1502 cm⁻¹ in the complexes, reflecting changes in electron distribution within the N–C–O moiety. These spectral shifts suggest the formation of a polarized resonance structure, where metal–oxygen coordination reduces the double-bond character of the C=O bond while enhancing the C–N bond order.^[15–17]^

Thermogravimetric analysis of the mixed precursor (Ni–urea + 2×Fe–urea) under oxidative conditions shows a rapid and substantial mass loss below ~200–250 °C, corresponding to the decomposition of urea ligands and nitrate species. The majority of organic components are removed at this temperature range, leaving a residual mass of approximately 15–20%. Beyond this stage, the mass remains relatively stable up to 1000 °C, indicating the formation of thermally stable inorganic residues. The XRD pattern of the residue obtained after TGA confirms the formation of a crystalline spinel NiFe₂O₄ phase, consistent with the expected decomposition pathway of metal–urea complexes. These results demonstrate that the coordinated precursors undergo efficient low-temperature decomposition, facilitating the formation of the desired oxide phase.

**Table S1.** List of samples prepared.

| Samples | Description | Tthermogravimetric analysis residual mass/ % | Crystallite size/Debye-Scherrer equation |
| --- | --- | --- | --- |
| Prisitne CNH | As purchased CNH | 0-1 | - |
| N-CNH | N-functionalized CNH | 0-1 | - |
| P-CNH | P-functionalized CNH | 0-1 | - |
| CNH-N-P | N and P functionalized CNH | 0-1 | - |
| NiFe_2_O_4_ | Pure nanoparticles | - | 18.5 nm |
| NiFe_2_O_4_@CNH | Nanohybrid of NiFe_2_O_4_ with pristine CNH | 51.4 | 8.5 nm |
| NiFe_2_O_4_@N-CNH | Nanohybrid of NiFe_2_O_4_ with N functionalized CNH | 56.6 | 8.3 nm |
| NiFe_2_O_4_@P-CNH | Nanohybrid of NiFe_2_O_4_ with P functionalized CNH | 54.1 | 7.1 nm |
| NiFe_2_O_4_@CNH-N-P | Nanohybrid of NiFe_2_O_4_ with N and P functionalized CNH | 55.8 | 8.2 nm |

**Figure S2.** Pure NiFe_2_O_4_ nanoparticles prepared by hydrothermal method using molecular precursors in stochiometric ratio, (a) XRD pattern shows major diffraction peaks at 18.2°, 30.3°, 35.7°, 37.3°, 43.4°, 53.9°, 57.4°, and 63.0°, well assigned to (111), (220), (311), (222), (400), (422), (511) and (440) planes of NiFe_2_O_4_ inverse spinel phase (JCPDS No. 74-2081), respectively, (b) TEM with inset histrogram of particle size distribution curve, (c) HRTEM, and (d) indexed SAED pattern.

**Figure S3**. TEM images of (a) prisitne CNH, (b) N-CNH, (c) P-CNH, and (d) CNH-N-P.

**Figure S4.** SAED pattern with indexed difraction peaks of the nanohybrids: (a) NiFe_2_O_4_@CNH, (b) NiFe_2_O_4_@N-CNH, (c) NiFe_2_O_4_@P-CNH, and (d) NiFe_2_O_4_@CNH-N-P.

**Figure S5.** Raman spectra of (a) pristine and functionalized CNHs and (b) all different ferrite/CNH nanohybrids.

**Table S2.** Band positions and integrated area fraction (I_D_/I_G_) for all samples.

| Samples | Raman bands / cm^−1^ | | | | I_D_/I_G_ (integrated area) |
| --- | --- | --- | --- | --- | --- |
|  | D | G | 2D | D+G |  |
| Pristine CNH | 1327 | 1583 | 2654 | 2899 | 1.64 |
| N-CNH | 1330 | 1583 | 2648 | 2907 | 1.74 |
| P-CNH | 1320 | 1581 | 2648 | 2900 | 1.91 |
| CNH-N-P | 1328 | 1591 | 2651 | 2902 | 1.99 |
| NiFe_2_O_4_@CNH | 1329 | 1586 | 2650 | 2901 | 1.66 |
| NiFe_2_O_4_@N-CNH | 1334 | 1591 | 2650 | 2920 | 1.85 |
| NiFe_2_O_4_@P-CNH | 1329 | 1586 | 2672 | 2906 | 1.88 |
| NiFe_2_O_4_@CNH-N-P | 1318 | 1578 | 2634 | 2874 | 1.91 |

**Table S3.** BET surface area and pore structural parameters of all samples.

| Samples | BET surface area/ m^2^/g | Average pore size /nm | Total pore volume/ cc/g | Micropore analysis/ t-plot method | |
| --- | --- | --- | --- | --- | --- |
|  |  |  |  | Micropore volume/ cc/g | Micropore area/ m^2^/g |
| CNH | 258.4 | 1.5 | 0.38 | 0.00 | 0.00 |
| N-CNH | 529.3 | 8.4 | 0.52 | 0.12 | 306.0 |
| P-CNH | 576.6 | 11.8 | 1.10 | 0.13 | 315.80 |
| CNH-N-P | 695.9 | 7.6 | 0.89 | 0.16 | 398.50 |
| NiFe_2_O_4_@CNH | 302.1 | 1.9 | 0.45 | 0.04 | 79.30 |
| NiFe_2_O_4_@N-CNH | 533.4 | 1.8 | 0.49 | 0.12 | 282.60 |
| NiFe_2_O_4_@P-CNH | 500.4 | 1.5 | 0.41 | 0.08 | 192.10 |
| NiFe_2_O_4_@CNH-N-P | 624.7 | 8.4 | 0.59 | 0.13 | 329.20 |
| CNH-N-P(2)  (synthesized by a single step treatment with phosphazene) | 412.5 | 11.6 | 0.94 | 0.07 | 212.10 |

**Figure S6.** XPS of pristine CNH and funcionalized CNHs. Survey spectra (a) and comperative plots of high resolution peaks of (b) C 1s, (c) N 1s (d) O1s and (e) P 2p.

**Table S4.** XPS generated elemental analysis of all the samples

| Samples | Elements/ (atomic %) | | | | | |
| --- | --- | --- | --- | --- | --- | --- |
|  | C | N | O | P | Ni | Fe |
| CNH | 85.9 | 1.0 | 13.2 | - | - | - |
| N-CNH | 90.5 | 2.4 | 2.6 | - | - | - |
| P-CNH | 97.4 | 1.2 | 1.5 | 0.5 | - | - |
| CNH-N-P | 84.2 | 2.2 | 3.8 | 2.3 | - | - |
| NiFe_2_O_4_@CNH | 81.8 | 5.8 | 9.4 | - | 1.1 | 2.1 |
| NiFe_2_O_4_@N-CNH | 83.0 | 5.5 | 8.3 | - | 1.2 | 2.0 |
| NiFe_2_O_4_@P-CNH | 81 | 4.8 | 9.9 | 0.4 | 1.3 | 2.6 |
| NiFe_2_O_4_@CNH-N-P | 50.2 | 4.1 | 29.4 | 1.6 | 4.4 | 11.1 |
| CNH-N-P(2)  (synthesized by a single step treatment with phosphazene) | 96.5 | 0.8 | 2.5 | 0.2 | - | - |

**Figure S7.** Survey spectra of NiFe_2_O_4_ NPs together with all ferrite/CNH nanohybrids. Minor signals of N and C in NiFe_2_O_4_ are due to remaining trace contaminations from the urea precursor route.

**Table S5.** XPS peak-fitted binding energies of CNH, CNH-N-P, NiFe_2_O_4_, and NiFe_2_O_4_@CNH-N-P. The observed binding-energy shifts (Δ eV) between the pristine components and the composite indicate electronic interaction and charge redistribution at the NiFe_2_O_4_@CNH-N-P interface.

| Core level fitted components | CNH  Peak BE (eV) | CNH-N-P  Peak BE (eV) | NiFe_2_O_4_  Peak BE (eV) | NiFe_2_O_4_@CNH-N-P  Peak BE (eV) | Shift (Δ eV) |
| --- | --- | --- | --- | --- | --- |
| P 2p_3/2_ (Peak 1) |  | 133.2 | - | 133.1 |  |
| P 2p_1/2_ (Peak 2) |  | 134.2 | - | 134.0 |  |
| C 1s (Peak 1) | 284.4 | 284.4 | 285.0 | 284.4 |  |
| C 1s (Peak 2) | 286.4 | 286.2 | 286.7 | 286.0 |  |
| C 1s (Peak 3) | 288.5 | 289.9 | 288.9 | 289.2 |  |
| C 1s (Peak 4) | 290.6 | - | - | - |  |
| N 1s (Peak 1) | 399.3 | 399.6 | 398.3 | 399.8 | +0.2 |
| N 1s (Peak 1) | 401.3 | 401.0 | 400.0 | - |  |
| O 1s (Peak 1) | - | - | 530.0 | 530.2 |  |
| O 1s (Peak 2) | 531.9 | 530.9 | 531.8 | 531.3 |  |
| O 1s (Peak 3) | 533.1 | 532.7 | 533.2 | 533.0 |  |
| Fe 2p_3/2_ (Peak 1) | - | - | 710.5 | 710.7 | +0.2 |
| Fe 2p_3/2_ (Peak 2) | - | - | 712.9 | 713.2 | +0.3 |
| Fe 2p_3/2_ (Peak 3) | - | - | 716.8 | 716.8 |  |
| Fe 2p_3/2_ (Peak 4) | - | - | 721.1 | 721.0 |  |
| Fe 2p_3/2_ (Peak 5) | - | - | 724.1 | 724.2 |  |
| Fe 2p_3/2_ (Peak 6) | - | - | 726.4 | 726.5 |  |
| Fe 2p_3/2_ (Peak 7) | - | - | 729.6 | 729.6 |  |
| Fe 2p_3/2_ (Peak 8) | - | - | 733.9 | 733.8 |  |
| Ni 2p_3/2_ (Peak 1) | - | - | 854.9 | 855.1 | +0.2 |
| Ni 2p_3/2_ (Peak 2) | - | - | 856.7 | 856.5 | -0.2 |
| Ni 2p_3/2_ (Peak 3) | - | - | 861.5 | 861.7 | +0.2 |
| Ni 2p_3/2_ (Peak 4) | - | - | - | 866.4 | - |

Δ eV is calculated as the binding energy of NiFe_2_O_4_@CNH-N-P relative to the corresponding standalone material (CNH-N-P for N 1s and NiFe_2_O_4_ for Ni and Fe core levels).

**Figure S8.** (a) SEM image of CNH-N-P and (b) corresponding EDX analysis along with elemental mapping images indicating elements C, O, N, and P evenly distributed.

**Figure S9.** (a) SEM image of NiFe_2_O_4_@CNH-N-P and (b) corresponding EDX analysis along with elemental mapping images indicating elements C, O, N, P, Ni, and Fe are homogeneously distributed.

**Figure S10.** Linear sweep voltammetry measurements of (a) CNH, (b) N-CNH, (c) P-CNH, and (d) CNH-N-P. All measurement were performed in O_2_ saturated 0.1 M KOH.

**Figure S11.** Linear sweep voltametery measurments of (a) NiFe_2_O_4_ NPs, (b) NiFe_2_O_4_@CNH, (c) NiFe_2_O_4_@N-CNH, (d) NiFe_2_O_4_@P-CNH, (e) NiFe_2_O_4_@CNH-N-P, and (f) 20% Pt/C. All measurement were performed in O_2_ saturated 0.1 M KOH.

**Table S6.** ORR characteristics for all the samples; half wave potential, onset potential, and limiting current density.

| Samples | Half wave potential/ V vs. RHE | Onset potential/ V vs. RHE | Diffusion limiting current density/ mA/cm^2^ | Tafel slope/ mV/dec | J/0.8 V | Mass activity (A/g)@0.8 V |
| --- | --- | --- | --- | --- | --- | --- |
| CNH | 0.63 | 0.72 | −3.0 | 178 | -0.081 | -0.16 |
| N-CNH | 0.66 | 0.76 | −3.7 |  | -0.052 | -0.080 |
| P-CNH | 0.69 | 0.79 | -4.8 |  | -0.032 | -0.053 |
| CNH-N-P | 0.71 | 0.83 | -5.2 | 88.9 | -0.122 | -0.2.3 |
| NiFe_2_O_4_ | 0.64 | 0.75 | -2.4 |  | -0.02 | -0.084 |
| NiFe_2_O_4_@CNH | 0.66 | 0.77 | -4.9 | 95.4 | -0.10 | -0.15 |
| NiFe_2_O_4_@N-CNH | 0.73 | 0.81 | −5.8 | 87.8 | -0.32 | -0.55 |
| NiFe_2_O_4_@P-CNH | 0.75 | 0.83 | -6.2 | 80.6 | -0.32 | -0.52 |
| NiFe_2_O_4_@CNH-N-P | 0.79 | 0.96 | −6.6 | 56.5 | -1.58 | -2.54 |
| 20% Pt/C | 0.80 | 0.88 | −5.4 | 91.4 | -2.05 | -3.36 |
| NiFe_2_O_4_@CNH-N-P(2) | 0.72 | 0.80 | −5.1 | - | - | - |
| Physical mixture: 44% NiFe_2_O_4_ and 56% CNH-N-P | 0.64 | 0.76 | −5.3 | - | - | - |

**Table S7.** RRDE measurement characteristics of all samples under study: number of electrons transferred and amount of peroxide formation (%).

| Samples | Number of electrons transferred / n | % H_2_O_2_ |
| --- | --- | --- |
| 20% Pt/C | 3.96 | 0.9 |
| CNH | 2.95 | 40.1 |
| N-CNH | 3.34 | 33.5 |
| P-CNH | 3.20 | 36.6 |
| CNH-N-P | 3.69 | 17.2 |
| NiFe_2_O_4_@CNH | 3.49 | 29.2 |
| NiFe_2_O_4_@N-CNH | 3.66 | 17.8 |
| NiFe_2_O_4_@P-CNH | 3.75 | 13.4 |
| NiFe_2_O_4_@CNH-N-P | 3.91 | 6.6 |

**Table S8.** OER data for all samples; onset potential, OER potential at current density of 10 mA/cm^2^ and overpotential required to attain 10 mA/cm^2^ of current density.

| Samples | Onset potential / V vs. RHE | OER potential@  j = 10 mA/cm^2^/ V vs. RHE | Overpotential (η) at 10 mA/cm^2^ /mv vs. RHE | Tafel slope  mV/ dec | Mass activity / A/g @ η = 250 mV |
| --- | --- | --- | --- | --- | --- |
| NiFe_2_O_4_ NPs | 1.69 | 1.85 | 620 | 119.2 | 0.21 |
| CNH-N-P | 1.62 | - |  | 150.2 | 0.38 |
| NiFe_2_O_4_@CNH | 1.55 | 1.60 | 370 | 102.5 | 3.30 |
| NiFe_2_O_4_@N-CNH | 1.54 | 1.59 | 360 | 85.3 | 3.10 |
| NiFe_2_O_4_@P-CNH | 1.50 | 1.55 | 320 | 81.7 | 6.22 |
| NiFe_2_O_4_@CNH-N-P | 1.45 | 1.52 | 290 | 79.9 | 6.77 |
| RuO_2_ | 1.43 | 1.49 | 260 | 76.4 | 13.80 |

**Figure S12.** CV curves of (a) CNH-P, (b) NiFe_2_O_4_@CNH, (c) NiFe_2_O_4_@N-CNH, (d) NiFe_2_O_4_@P-CNH, (e) NiFe_2_O_4_@CNH-N-P (f) 20% Pt/C+RuO_2_ at different scan rates, (g) fitted current densities against scan rates to determine electrochemical double-layer cappitance (C_dl_), (h) Nyquist plot from EIS measurements, and (i) table list of charge tranfer constant, corresponding ECSA and R_ct_ values. All the measuements were performed in 0.1 M KOH.

**Figure S13**. Intrinsic bifunctional electrocatalytic activity comparison normalized by catalyst mass loading (0.5 mg/cm^2^). (a) ORR mass activity linear sweep voltammetry (LSV) polarization curves of pristine and heteroatom-doped carbon nanohorns (CNH). (b) ORR mass activity LSV curves of NiFe_2_O_4_ based hybrid electrocatalysts compared with commercial 20% Pt/C. (c) OER mass activity polarization curves demonstrating the intrinsic performance of all studied catalysts relative to commercial RuO_2_. (d) Comparative bar plot summarizing the geometric current density (orange, J_mA/cm_^2^) and mass activity (green, A/g) at a fixed potential of 0.8 V vs. RHE for ORR across all samples. All measurements were performed in O_2_-saturated 0.1 M KOH; catalyst mass loading on the working/rotating disk electrode was 0.5 mg/cm^2^. Mass activity was calculated as j/m, where j is the measured current density at the specified potential and m is the catalyst mass loading per unit geometric electrode area.

**Figure S14.** CNH-N-P(2) functionalized by single step method using a cyclotriphosphazene compound. (a) survey spectra CNH-N-P(2), (b) C 1s, (c) N 1s, (d) P 2p, (e) comparision of Isotherm and pore size distribution characteristics of CNH-N-P and CNH-N-P(2), and (f) ORR comparison plots (1600 rpm) of CNH-N-P, CNH-N-P(2), NiFe_2_O_4_@CNH-N-P, NiFe_2_O_4_@CNH-N-P(2).

**Figure S15**. (a) ORR polarization curves of pure NiFe_2_O_4_, CNH-N-P, a physical mixture of the two 44 wt.% NiFe_2_O_4_ + 56 wt.% CNH-N-P (matched to the TGA-determined composition of NiFe_2_O_4_@CNH-N-P) and the synthesized NiFe_2_O_4_@CNH-N-P nanohybrid. Post-cycling structural and surface characterization of the NiFe_2_O_4_@CNH-N-P catalyst: (b,c) High-resolution XPS spectra of Ni 2p and Fe 2p for the pristine electrode, and washed and unwashed after the zinc-air battery cycled electrode. (d,e) SEM-EDX spectra and quantitative elemental analysis of the catalyst electrode before and after cycling. (f) HRTEM image of the catalyst after cycling (3 cycles), the inset shows lattice fringes assigned to the planes of spinel NiFe_2_O_4_. XPS and SEM-EDX characterization was conducted after the claimed cycle life of the battery, while TEM/HRTEM characterization was performed on an electrode recovered after 3 cycles.

**Table S9.** Discharge capacity, Depth of dischage and maximum number of glavanostatic cyclic activity of nanohybrid samples

| Samples | Maximum Discharge capacity /mAh/g | Depth of discharge/ % | Number of cycles |
| --- | --- | --- | --- |
| NiFe_2_O_4_@CNH | 187.6 | 22.8 | 31 |
| NiFe_2_O_4_@N-CNH | 272.6 | 33.24 | 46 |
| NiFe_2_O_4_@P-CNH | 286.70 | 34.94 | 53 |
| NiFe_2_O_4_@CNH-N-P | 293.50 | 35.8 | 120 |
| 20% Pt/C+RuO_2_ | 264.4 | 32.2 | 17 |

**Figure S16.** Specific discharge capacity versus cycle number plot of nanohybrids compared to 20% Pt/C+RuO_2_.

**Table S10**. ICP-OES analysis of the electrolyte before and after long term charge-discharge cycling test.

| Elements | Fresh 4 mol/L KOH electrolyte | Post cycling 4 mol/L KOH electrolyte |
| --- | --- | --- |
| Ni | Undetected (< 0.0 μg/ L | Undetected (< 0.0 0.0 μg/ L |
| Fe | 0.259 mg/ L | 0.259 mg/ L |

**Table S11.** Summary of NiFe_2_O_4_/carbon composites reported in the literature.

| Catalyst architecture | ORR E_1/2_ (V vs. RHE) | OER Overpotential η_10_ (mV) | Bifunctional Gap ΔE (V) | ZAB Stability / Cycling | Specific capacity with respect to consumed zinc | Reference |
| --- | --- | --- | --- | --- | --- | --- |
| NiFe_2_O_4_@CNH-N-P | 0.79 | 290 | 0.73 | 300 h+ (ZnO/C), 250 h (Zn foil) | 763_(consumed Zn)_,  293.5 _(ZnO/C)_ | Present work |
| NiFe_2_O_4_(QDs)/CNTs | - | - | 0.90 | 200 h | 801 | ^[18]^ |
| NiFe_2_O_4_/N-CNT | 0.84 | 313 | 0.70 | 101.5 h | 798 | ^[19]^ |
| NiFeOx@VACNTs | - | - | 0.74 | 60 h | 800 | ^[20]^ |
| FeNi/NiFe_2_O_4_@N-carbon sponge | 0.84 V | 370 | 0.76 | 80 h | 837 | ^[21]^ |
| MoSe_2_@NiFe_2_O_4_/Vulcan carbon | 0.68 | 370 | - | 150 h | - | ^[22]^ |
| Fe_2_Ni_1_/NiFe_2_O_4_@Graphene oxide | 0.83 | 310 | 0.61 | 100 h | 770 | ^[23]^ |
| NiFe_2_O_4_/N-doped graphene | 0.85 | 294 | 0.67 | 20 h | 513.6 | ^[24]^ |
| NiFe_2_O_4_/Bamboo-Like Carbon Nanotubes | 0.88 | 300 | - | 200 h | - | ^[25]^ |

**References**

[1]	K. Veldboer, Y. Karatas, T. Vielhaber, U. Karst and H.-D. Wiemhöfer, "Cyclic Phosphazenes for the Surface Modification of Lanthanide Phosphate‐based Nanoparticles," *Zeitschrift für anorganische und allgemeine Chemie* 634, no. 12-13 (2008): 2175. https://doi.org/10.1002/zaac.200800297.

[2]	T. Bies, R. C. Hoffmann, M. Stöter, A. Huber and J. J. Schneider, "Environmentally Benign Solution‐Based Procedure for the Fabrication of Metal Oxide Coatings on Metallic Pigments," *Chemistry Open* 1251–1263: 1251.

[3]	T. Bies, "Thermische Degradation von Metallkomplexen zur lösungsbasierten Oxidbeschichtung von Aluminiumpigmenten," *Thesis (*2021).

[4]	J. H. Scofield, "Hartree-Slater subshell photoionization cross-sections at 1254 and 1487 eV," *Journal of Electron Spectroscopy and Related Phenomena* 8 (1976): 129.

[5]	K. Xiao, Y. Wang, P. Wu, L. Hou and Z.-Q. Liu, "Activating Lattice Oxygen in Spinel ZnCo_2_O_4_ through Filling Oxygen Vacancies with Fluorine for Electrocatalytic Oxygen Evolution," *Angewandte Chemie International Edition* 62, no. 24 (2023): e202301408.

[6]	S. Niu, S. Li, Y. Du, X. Han and P. Xu, "How to Reliably Report the Overpotential of an Electrocatalyst," *ACS Energy Letters* 5, no. 4 (2020): 1083. https://doi.org/10.1021/acsenergylett.0c00321.

[7]	A. Vignesh, M. Prabu and S. Shanmugam, "Porous LaCo_1-x_Ni_x_O_3-δ_ Nanostructures as an Efficient Electrocatalyst for Water Oxidation and for a Zinc-Air Battery," *ACS applied materials & interfaces* 8, no. 9 (2016): 6019. https://doi.org/10.1021/acsami.5b11840.

[8]	T. Naresh Kumar, S. Sivabalan, N. Chandrasekaran and K. L. Phani, "Synergism between polyurethane and polydopamine in the synthesis of Ni-Fe alloy monoliths," *Chemical communications* 51, no. 10 (2015): 1922. https://doi.org/10.1039/c4cc07534j.

[9]	M. Luo, Z. Zhao and Y. Zhang et al., "PdMo bimetallene for oxygen reduction catalysis," *Nature* 574, no. 7776 (2019): 81. https://doi.org/10.1038/s41586-019-1603-7.

[10]	Jin Suntivich, Kevin J. May, Hubert A. Gasteiger, John B. Goodenough, Yang Shao-Horn, "A Perovskite Oxide Optimized for Oxygen Evolution Catalysis from Molecular Orbital Principles," *Science (New York, N.Y.)* 334, no. 6061 (2011): 1383. https://doi.org/10.1126/science.1211927.

[11]	D. Deckenbach and J. J. Schneider, "A 3D hierarchically porous nanoscale ZnO anode for high-energy rechargeable zinc-air batteries," *Journal of Power Sources* 488 (2021): 229393. https://doi.org/10.1016/j.jpowsour.2020.229393.

[12]	Y. Zhou, R. Lu and X. Tao et al., "Boosting Oxygen Electrocatalytic Activity of Fe-N-C Catalysts by Phosphorus Incorporation," *Journal of the American Chemical Society* 145, no. 6 (2023): 3647. https://doi.org/10.1021/jacs.2c12933.

[13]	J. F. Parker, J. S. Ko, D. R. Rolison and J. W. Long, "Translating Materials-Level Performance into Device-Relevant Metrics for Zinc-Based Batteries," *Joule* 2, no. 12 (2018): 2519. https://doi.org/10.1016/j.joule.2018.11.007.

[14]	Y. Meng, Y.-M. Zhao and J.-C. Li et al., "An integrated oxygen electrode derived from a flexible single-walled carbon nanotube film for rechargeable Zn-air batteries produced by electropolymerization," *NPG Asia Materials* 15:14, no. 1 (2023): 1. https://doi.org/10.1038/s41427-022-00441-0.

[15]	T. Bies, R. C. Hoffmann, M. Stöter, A. Huber and J. J. Schneider, "Environmentally Benign Solution-Based Procedure for the Fabrication of Metal Oxide Coatings on Metallic Pigments," *ChemistryOpen* 9, no. 12 (2020): 1251. https://doi.org/10.1002/open.202000223.

[16]	Y. Akamine, F. Nishino and T. Yamashita et al., "The Impact of Metal-Support Interaction on the Structure and Activity of Carbon-Supported Ni Nanoparticle Catalysts for Alkaline Hydrogen Oxidation Reaction," *ACS applied materials & interfaces* 16, no. 50 (2024): 69316. https://doi.org/10.1021/acsami.4c15120.

[17]	O. Carp, L. Patron, L. Diamandescu, A. Reller, "Thermal decomposition study of the coordination compound [Fe(urea)_6_](NO_3_)_3_," *Thermochimica Acta* 390 (2002): 167.

[18]	N. Xu, Y. Zhang, T. Zhang, Y. Liu and J. Qiao, "Efficient quantum dots anchored nanocomposite for highly active ORR/OER electrocatalyst of advanced metal-air batteries," *Nano Energy* 57 (2019): 176. https://doi.org/10.1016/j.nanoen.2018.12.017.

[19]	Y. Tang, Y. Lei and G. Li et al., "Positive regulation of active sites for oxygen evolution reactions by encapsulating NiFe_2_O_4_ nanoparticles in N-doped carbon nanotubes in situ to construct efficient bifunctional oxygen catalysts for rechargeable Zn–air batteries," *Journal of Materials Chemistry A* 10, no. 10 (2022): 5305. https://doi.org/10.1039/D1TA10881F.

[20]	Y. Yan, Y. Xu and B. Zhao et al., "Bifunctional nickel ferrite-decorated carbon nanotube arrays as free-standing air electrode for rechargeable Zn–air batteries," *Journal of Materials Chemistry A* 8, no. 10 (2020): 5070. https://doi.org/10.1039/D0TA00554A.

[21]	H. Wang, S. Su and T. Yu et al., "FeNi/NiFe_2_O_4_ hybrids confined in N-doped carbon sponge derived from Hofmann-type MOFs for oxygen electrocatalysis," *Applied Surface Science* 596 (2022): 153522. https://doi.org/10.1016/j.apsusc.2022.153522.

[22]	M. M. Sebastian, D. Dixon, T. D. Thangadurai, N. Kalarikkal and A. Schechter, "NiFe_2_O_4_ in MoSe_2_ Exhibits Bifunctional Water Oxidation and Oxygen Reduction (OER and ORR) Catalytic Reactions for Energy Applications," *ACS Applied Energy Materials* 7, no. 19 (2024): 8635. https://doi.org/10.1021/acsaem.4c01586.

[23]	Y. Chen, G. Li and L. Hong et al., "FeNC Catalysts Decorated with NiFe_2_O_4_ to Enhance Bifunctional Activity for Zn–Air Batteries," *ACS Applied Energy Materials* 6, no. 11 (2023): 6357. https://doi.org/10.1021/acsaem.3c00942.

[24]	J.-Y. Qin, S. Wang, S. Zhou, T. Liu, Y. Yin and J. Yang, "Dominating Role of Interfacial N–Ni Coordination in Spinel Nickel Ferrite/N‐Doped Graphene Hybrids for Boosting Reversible Oxygen Electrocatalysis," *Advanced Energy and Sustainability Research* 2, no. 5 (2021): 2000106/1-2000106/8. https://doi.org/10.1002/aesr.202000106.

[25]	M. Wang, S. Ji, H. Wang, X. Wang, V. Linkov and R. Wang, "Foamed Carbon-Supported Nickel-Iron Oxides Interspersed with Bamboo-Like Carbon Nanotubes for High-Performance Rechargeable Zinc-Air Batteries," *Small,* 18, no. 45 (2022): 2204474. https://doi.org/10.1002/smll.202204474.
